# Synergistic Therapeutic Platform Combining Transcranial Low-Intensity Ultrasound Stimulation and Curcumin Load Liposome Ameliorates Cerebral Ischemia by Modulating Microglia Polarization-Mediated Neuroinflammatory Microenvironment

**DOI:** 10.34133/research.0861

**Published:** 2025-09-11

**Authors:** Haocheng Qin, Di Chen, Bao Zhou, Pengkun Yang, Ya Zheng, Lu Sun, Zhengran Ding, Zhong He, Shuai Zhang, Zijian Hua, Gang Chen, Zhiwen Luo, Yulian Zhu, Yi Wu

**Affiliations:** ^1^ Department of Rehabilitation, Huashan Hospital, Fudan University, Shanghai, China.; ^2^ Department of Neurosurgery, The First Affiliated Hospital of Zhengzhou University, Zhengzhou University, Zhengzhou, Henan, China.; ^3^ Department of Orthopaedics, Jiaxing Key Laboratory of Basic Research and Clinical Translation on Orthopedic Biomaterials, The Second Affiliated Hospital of Jiaxing University, Jiaxing 314000, China.; ^4^ Department of Sports Medicine, Huashan Hospital, Fudan University, Shanghai, China.

## Abstract

Successful restoration of blood supply still follows with serious secondary brain injury in cerebral ischemia reperfusion injury (CIRI). However, conventional management after CIRI exhibits numerous limitations.Transcranial low-intensity ultrasound stimulation (TLUS) has been extensively utilized in the field of neuromodulation owing to its characteristics of zero potential side effects, noninvasiveness, deep tissue penetration, and precise spatial targeting capability. Here, we develop a smart multifunctional ultrasound-responsive delivery system to regulate CIRI. Briefly, curcumin, a well-documented safe neuroprotective monomer derived from traditional Chinese medicine, is encapsulated into self-assembled liposomal nanocarriers (RRP@Lipo-Cur). These nanocarriers are modified with reactive oxygen species- and ultrasound-responsive functional elements, as well as neuron-targeting peptides, enabling targeted accumulation in ischemic penumbra and stimuli-responsive drug release. In the mouse model of middle cerebral artery occlusion/reperfusion (MCAO/r), the synergistic treatment of RRP@Lipo-Cur and TLUS significantly facilitated the polarization of microglia from the M1 to the M2 phenotype via the intracellular mitogen-activated protein kinase (MAPK) and nuclear factor κB signaling (NF-κB) pathways, which effectively promoted the establishment of an anti-inflammatory microenvironment in the ischemic penumbra, thereby providing neuroprotection for the damaged neurons. Furthermore, in comparison to single nanomaterial or TLUS interventions, synergistic treatment can optimally modulate neuroinflammation, safeguard injured neurons, and enhance the recovery of neurological function.

## Introduction

Stroke has emerged as a major worldwide health issue due to its high mortality and disability [1]. Ischemic stroke constitutes around 80% of all stroke cases and happens when the cerebral arteries that ensure adequate blood flow to the brain become obstructed [2]. Opening an occluded artery within the therapeutic time window is considered the gold standard treatment for ischemic stroke [3]. Two primary strategies are tissue plasminogen activator thrombolytic therapy and endovascular thrombectomy [4]. However, successful restoration of blood supply still follows with serious secondary brain injury, including extensive neuroinflammation, disruption of the blood–brain barrier (BBB), neuron apoptosis, uncontrollable oxidative stress [5], and so on [6]. Ameliorating these pathophysiological changes accompanied by ischemia–reperfusion injury has become a priority in the treatment of ischemic stroke [7].

Development of traditional neuroprotective agents used for rehabilitative period faces multiple challenges associated with its limited bioavailability, potential side effects, limited targeted ability, rapid metabolism, and poor BBB penetration [8]. Therefore, there is a pressing need for the development of an efficient and targeted therapeutic strategy to address cerebral ischemia reperfusion injury (CIRI) [9].

As a physical modulatory factor, transcranial low-intensity ultrasound stimulation (TLUS) has been widely applied in the neuromodulation field due to its characteristics of zero potential side effects, noninvasiveness [10], deep penetration [11], and precise space targeting capability [12]. Furthermore, numerous studies on the central nervous system (CNS) have confirmed that TLUS could be regarded as a trigger [13] to release nanobiomaterials to specific regions of the brain in a controllable, noninvasive manner while also encouraging the BBB opening [14]. Besides, the combined treatment mode of continuous TLUS irradiation at the lesion with the released agents is also worthy of further investigation [15]. In this study, we utilized protoporphyrin IX (PpIX) as a sonosensitizer [16] for remotely triggered drug delivery as its ultra-high potency and minimal toxicity [17].

Curcumin, a proven safe neuroprotective traditional Chinese medicine monomer for cerebral ischemia, is an active ingredient in the rhizome of curcuma longa, a plant belonging to the ginger family, with the chemical formula C_21_H_20_O_6_ [18]. Curcumin has been widely reported to mitigate ischemic stroke by regulating multiple pathophysiological processes [19], including suppressing overactive neuroinflammation, protecting from neuron apoptosis and pyroptosis [20], mediating oxidative stress, and so on [21]. Nonetheless, poor absorption, low water solubility, rapid metabolism, and pharmacokinetic limitations constrain the clinical utilization of curcumin, which finally hinder its ability to fully reach the ischemic and hypoxic regions of the brain to optimize its therapeutic efficacy [22]. Delivering curcumin to the targeted location is a significant hurdle for its therapeutic application during CIRI treatment and constitutes an urgent necessity [23]. Liposomes, frequently employed as drug delivery vehicles in functional nanobiomaterials, can address the aforementioned issues by improving curcumin solubility and steadiness while integrating targeted delivery and prolonged circulation strategies to markedly increase the bioavailability of therapeutic agent to the infarct region of ischemic stroke [24]. The rabies virus glycoprotein peptide (RVG) is a glycoprotein defined by a 29-amino acid sequence present on the surface of the rabies virus, which selectively binds to nicotinic acetylcholine receptors (nAChRs) on neuronal cells [25]. Research indicates that RVG29 ligand-modified nanomaterials can effectively traverse the BBB, positioning the RVG29 peptide as an effective modification for CNS-targeted drug delivery [26]. Besides, reactive oxygen species (ROS) increase during cerebral ischemia is a hallmark pathophysiological change of ischemic stroke. As a subclass of the thioether group, thioketal (TK) is thought to be biodegradable, nontoxic, and suitable to integration into polymers or conjugates. When exposed to ROS, it cleaves to a thiol-containing group and acetone [27]. Thus, TK could act as a functional group to trigger drug release at the site of cerebral ischemia [28].

In this study, we developed a neuron-targeted ROS- and ultrasound-responsive liposomal drug delivery system and encapsulated with curcumin (RRP@Lipo-Cur). This smart nanoplatform possesses the functions of CNS targeting, intelligent release of curcumin to ischemic brain regions, controllable release of curcumin after TLUS stimulation, improving the pharmacokinetic limitations of curcumin, and collaborative therapy model of curcumin and TLUS.

**Fig. 1.**
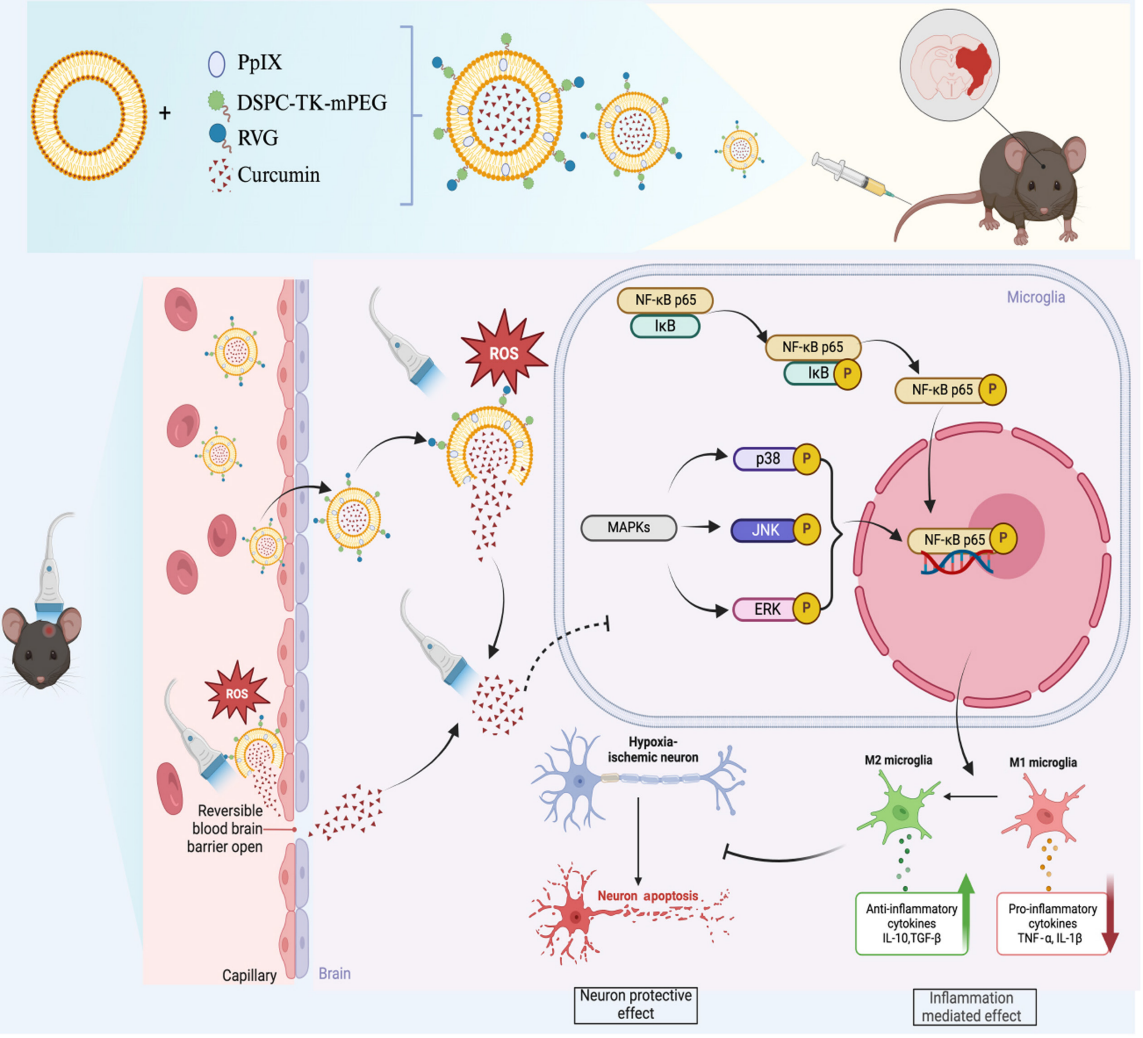
Illustration of mechanism of TLUS + RRP@Lipo-Cur synergistic therapy in CIRI. Schematic illustration of therapeutic mechanisms. Firstly, the RVG29 peptide facilitated the accumulation of RRP@Lipo-Cur in the brain. Then, the drug crosses the BBB through RVG peptide-modified liposomal carriers, and the reversible BBB opening is mediated by TLUS, subsequently accumulating in the ischemic brain. Moreover, the BBB opening induced by TLUS also enhanced the permeation of curcumin that had already been released in the bloodstream. RRP@Lipo-Cur triggers curcumin release under the dual effects of 1,2-distearoyl-sn-glycero-3-phosphoethanolamine (DSPE)-thioketal (TK)-methoxy-polyethylene glycol (mPEG)- and protoporphyrin IX (PpIX)-induced liposomal disintegration in the ROS-enriched microenvironment and TLUS activation. With the combined treatment of TLUS and curcumin, the modulation of the MAPK signaling pathway and NF-κB signaling pathway promotes the polarization of microglia from the M1 proinflammatory phenotype toward the M2 anti-inflammatory phenotype, improving extracellular inflammatory microenvironment, while simultaneously exerting neuroprotective effects.

This study preliminarily proves that smart liposomes not only effectively target ischemic brain regions, promoting curcumin retention, but also promote intelligent release of curcumin with stimulation of TLUS and high-level ROS microenvironment. In addition to a key role in the controlled release of curcumin, TLUS’s collaborative treatment with curcumin modulates expression of inflammatory cytokines by regulating the polarization of microglia through nuclear factor κB (NF-κB) and mitogen-activated protein kinase (MAPK) signaling pathways. The changed inflammatory microenvironment further inhibited the apoptosis of hypoxic–ischemic neurons (Fig. 1). In summary, this research presents a promising concept that integration of targeted drug delivery nanomaterials with TLUS creates a synergistic therapeutic system, through which targeted drug delivery, intelligent drug release, and the collaborative therapy of TLUS with therapeutic agents were effectively integrated to ensure an optimal therapeutic effect on cerebral ischemia.

## Results

### Preparation and characterization of RRP@Lipo-Cur

**Fig. 2.**
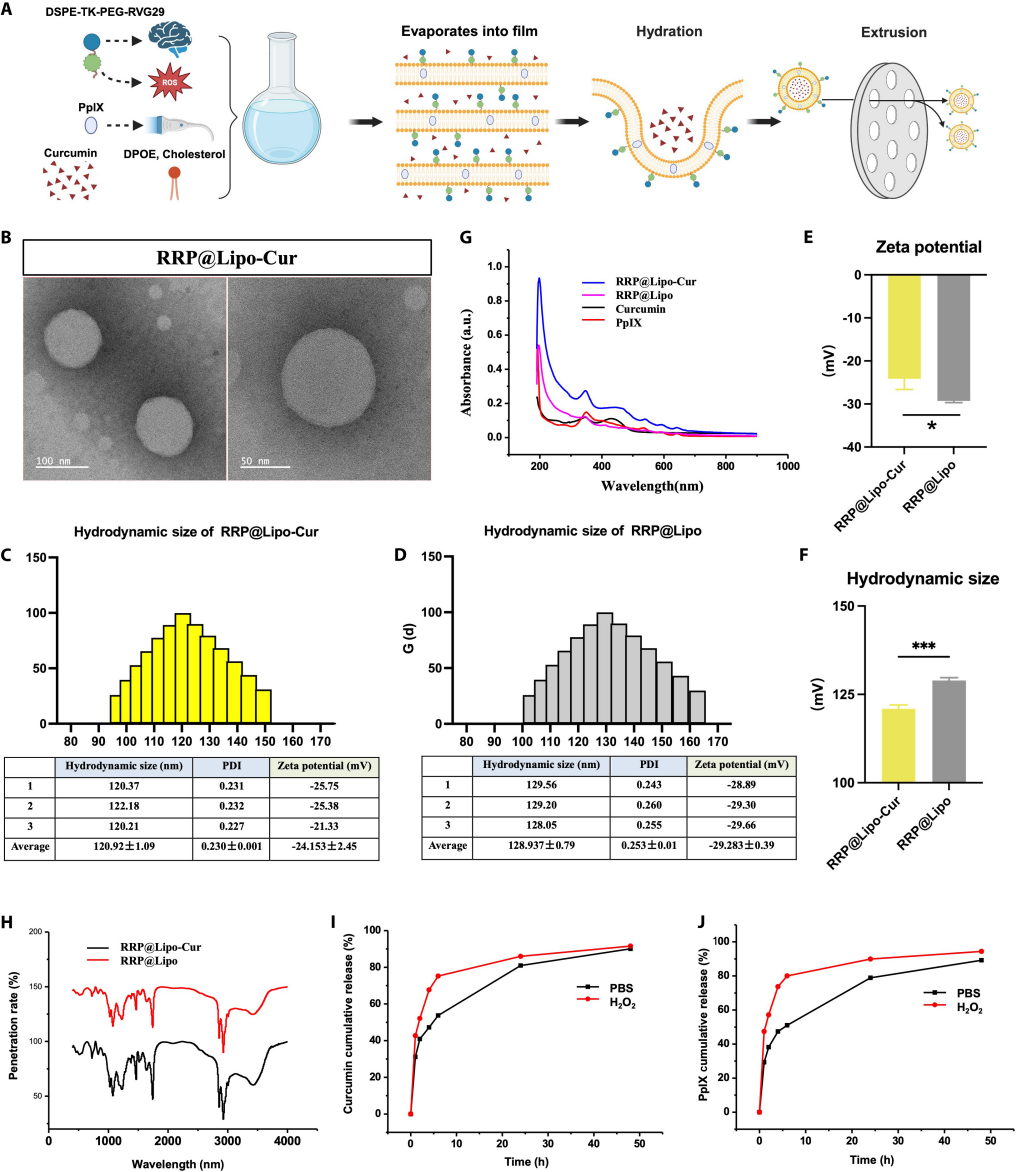
Preparation and characterization of RRP@Lipo-Cur. (A) Flowchart of preparation of RRP@Lipo-Cur. (B) TEM image of RRP@Lipo-Cur liposomes (scale bars, 100 nm and 50 nm). (C and D) Hydrodynamic size distribution and ζ-potential of RRP@Lipo-Cur and RRP@Lipo liposomes (*n* = 3). (E) Chart statistics of ζ-potential of RRP@Lipo-Cur and RRP@Lipo (*n* = 3). *P < 0.05. (F) Chart statistics of hydrodynamic size of RRP@Lipo-Cur and RRP@Lipo (*n* = 3). ***P < 0.001. (G) UV-Vis spectra of RRP@Lipo-Cur, RRP@Lipo, curcumin, and PpIX. (H) Infrared spectrum of RRP@Lipo-Cur and RRP@Lipo. (I and J) The cumulative release of curcumin and PpIX from RRP@Lipo-Cur in H_2_O_2_ and PBS from 0 to 50 h.

The method of thin-film hydration was applied to establish curcumin-loaded ROS-responsive RVG29 and PpIX-modified liposomes (RRP@Lipo-Cur) with 1,2-dioleoyl-sn-glycero-3-phosphoethanolamine (DOPE)/cholesterol/DSPE-TK-mPEG-RVG29 at a ratio of 200 mg:16 mg:16 mg in 5 ml of chloroform and 15 mg of PpIX and 8 mg of curcumin in methanol. Blank RRP@Lipo were established in the same way but without loading curcumin. These 2 liposomes were characterized as follows. As shown in transmission electron microscopy (TEM) images, the morphology of RRP@Lipo-Cur was uniformly sized in spherical morphology (Fig. 2B). The particle sizes of blank RRP@Lipo and RRP@Lipo-Cur were about 128.937 ± 0.79 nm and 120.92 ± 1.09 nm, respectively, as measured by dynamic light scattering (Fig. 2C and D). Figure 2F shows that there is a significant difference of particle between RRP@Lipo and RRP@Lipo-Cur, indicating the successful loading of curcumin. To ensure uniformity in nanoparticle size, we used a liposome extruder (Mini-extruder, Avanti, USA) to pass through the polycarbonate membrane (100 nm, Whatman, USA) 3 times. Besides, to ensure its stability in blood circulation and cell culture medium, the particle size was additionally detected at 24, 48, and 72 h after dissolving in phosphate-buffered saline (PBS), and the results showed that there was no significant change in the particle size at these 3 time periods, which indicate that the nanoparticle has stable characteristics (Fig. S3). The ζ-potentials of blank RRP@Lipo and RRP@Lipo-Cur were −29.283 ± 0.39 mV and −24.153 ± 2.45 mV, respectively. Results of ζ-potentials additionally demonstrated the successful curcumin loading, but it did not greatly affect physical and chemical features of liposomes (Fig. 2E). Negative surface charge, on the other hand, helped prevent nonspecific phagocytosis in vivo. In addition, encapsulation efficiency (EE) and drug loading capacity (LC) of curcumin were 61.2% and 4.49%, respectively, while the EE and LC of PpIX were 62.3% and 4.57%, respectively. The expression of absorbance at each wavelength also showed the successful construction of RRP@Lipo-Cur and its composition presented by ultraviolet–visible (UV-Vis) spectra (Fig. 2G). Infrared spectrum analysis in Fig. 2H presented the different penetration rate of the nanoparticle before and after curcumin loading, which indicated successful encapsulation of curcumin. Besides, Fig. 2H also demonstrated that the structural properties of the nanoparticles did not change significantly after loading curcumin, indicating that curcumin loading did not affect the integrity of the nanoparticle. However, it is our limitation that we fail to determine the substance represented by a certain band or peak with our current technology. To verify the ROS-responsive properties of the liposomes, the cumulative release efficiency of curcumin as well as PpIX in PBS and 5 mM H_2_O_2_ liquid environments was examined. The result demonstrated that the release efficiency of curcumin was significantly increased in the condition of H_2_O_2_ environment at different time points in the first 24 h (Fig. 2I and Fig. S4A to F), while the PpIX release was also significantly increased at each time point during 48 h when exposed to H_2_O_2_ (Fig. 2J and Fig. S4G to L). The cumulative release curve also indicated that the stability of RRP@Lipo-Cur supported the further use in animals. To exclude the potential therapeutic effects of the carrier itself on ischemia–reperfusion injury, we administered RRP@Lipo to treat middle cerebral artery occlusion (MCAO) mice. Western blot (WB) and 2,3,5-triphenyltetrazolium chloride (TTC) staining results indicated that the unloaded nanomaterial group did not significantly exhibit neuroprotective effects (Fig. S7A to F).

### Application of TLUS

**Fig. 3.**
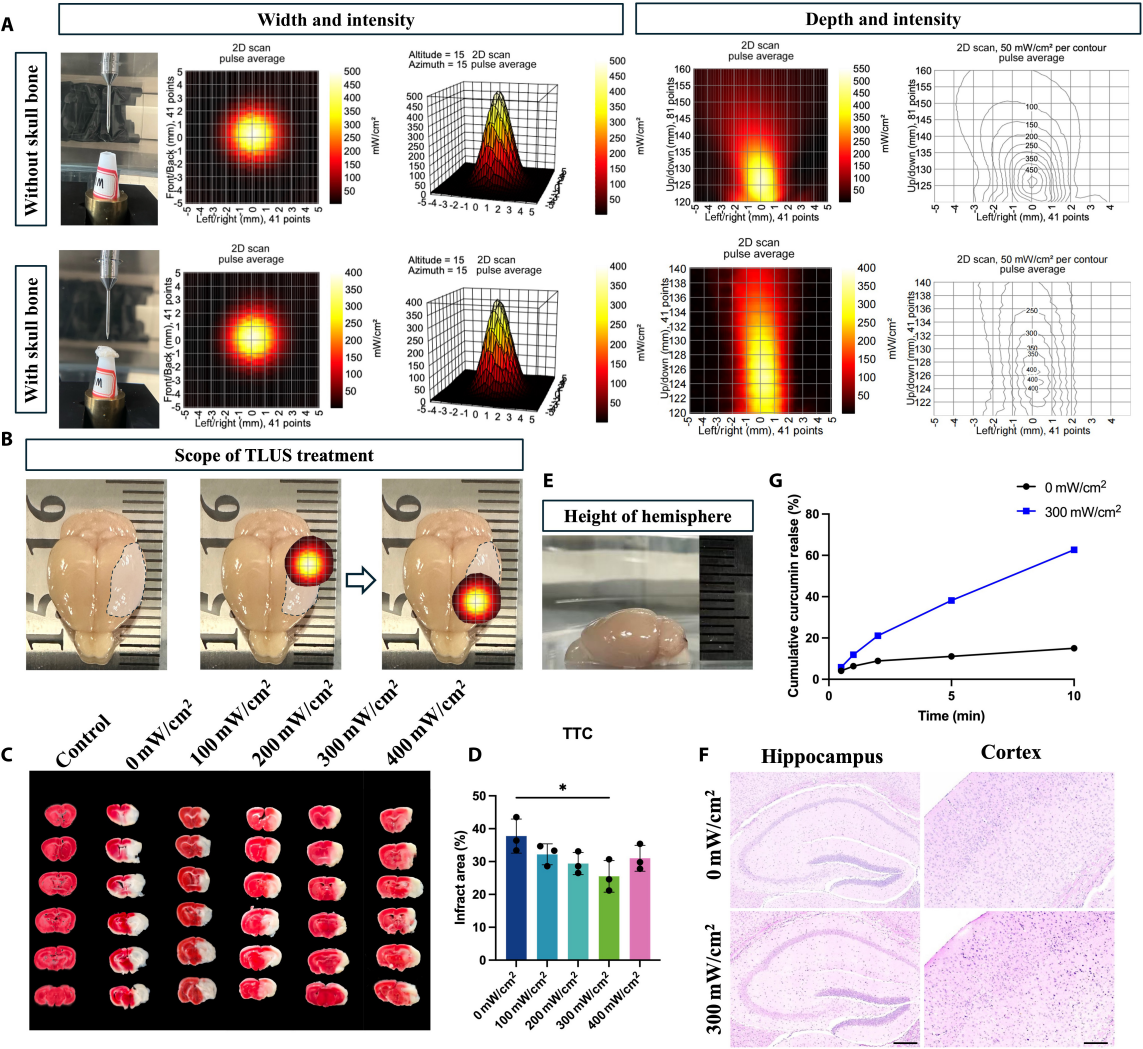
Application of TLUS. (A) Average pulse analysis of ultrasound effects on without skull and with skull mouse brains in width–intensity and depth–intensity correlations under 2D scanning. (B) Scope of TLUS treatment on the mouse brain, and the annular markers denote the treatment sites. (C) TTC staining comparing effects of different “brain parenchyma intensity” (0, 100, 200, 300, and 400 mW/cm^2^) on cerebral infarction (white, infarcted regions; red, normal tissue). (D) The infarct areas across groups were analyzed (*n* = 3). *P < 0.05. (E) Cerebral hemisphere height measurement in mice. (F) Representative H&E staining images of hippocampal and cortical regions in 2 groups treated with different ultrasound intensities (0 and 300 mW/cm^2^). Scale bar, 250 μm. (G) Cumulative release of curcumin from RRP@Lipo-Cur under ultrasound exposure at 0 and 300 mW/cm^2^.

First, to ascertain the shielding impact of the skull on sound transmission, we used a hydrophone (ONDA, USA) to detect the variations in intensity and irradiation range of the ultrasonic transducer with and without the mouse skull. The results showed that the magnitude of ultrasound intensity decreased significantly after passing through the skull. The intensity in peak zone decreased from 500 mW/cm^2^ to 400 mW/cm^2^. Here, we defined these 2 intensities as “transducer output intensity” and “brain parenchyma intensity”, respectively (Fig. 3A). The depth was also reduced from 12.5 mm to 10 mm, while the width of the effective area was not obviously reduced, maintaining at 4 mm^2^ (Fig. 3A). A depth of 10 mm effectively covered the affected hemisphere of the mouse (Fig. 3D). However, the output area of 4 mm^2^ could not completely cover the infarcted brain area. Therefore, in the treatment of MCAO/r mice, the anterior and posterior hemispheres of the infarcted zone were treated for 5 min each (Fig. 3B). The depth of effect of TLUS extends beyond that of the cerebral hemisphere, indicating that the stimulation depth is adequate for the intended application (Fig. 3E). The treatment procedure was shown in Fig. S5. To obtain the optimal stimulation intensity, we used different gradients of brain parenchyma intensity to treat MCAO/r mice. According to TTC findings, 300 mW/cm^2^ of brain parenchyma intensity showed the most significant reduction in infarct volume compared to other gradients of intensity (Fig. 3C and D). Thus, 300 mW/cm^2^ of brain parenchyma intensity was used as standard intensity for the following TLUS intervention. Hematoxylin and eosin (H&E) staining results of the cortex and hippocampus after TLUS treatment (2 times, 5 min, 300 mW/cm^2^) showed no distinct pathological changes (Fig. 3F). Last, we used UV spectrophotometry to determine the rate of curcumin release of RRP@Lipo-Cur with different durations (30 s, 1 min, 2 min, 5 min, and 10 min) at 300 mW/cm^2^ transducer output intensity in vitro. The results showed that ultrasound irradiation significantly promoted the release of curcumin (Fig. 3G). Meanwhile, apoptosis flow cytometry was performed to investigate the effect of 300 mW/cm^2^ transducer output intensity on HT-22 cell viability. The results revealed that HT-22 cells stimulated at this intensity for 10 min did not exhibit considerable cell apoptosis (Fig. S6). The safety of this intensity was verified by both in vivo and in vitro experiments.

### Biotargeting capability of RRP@Lipo-Cur both in vitro and in vivo

**Fig. 4.**
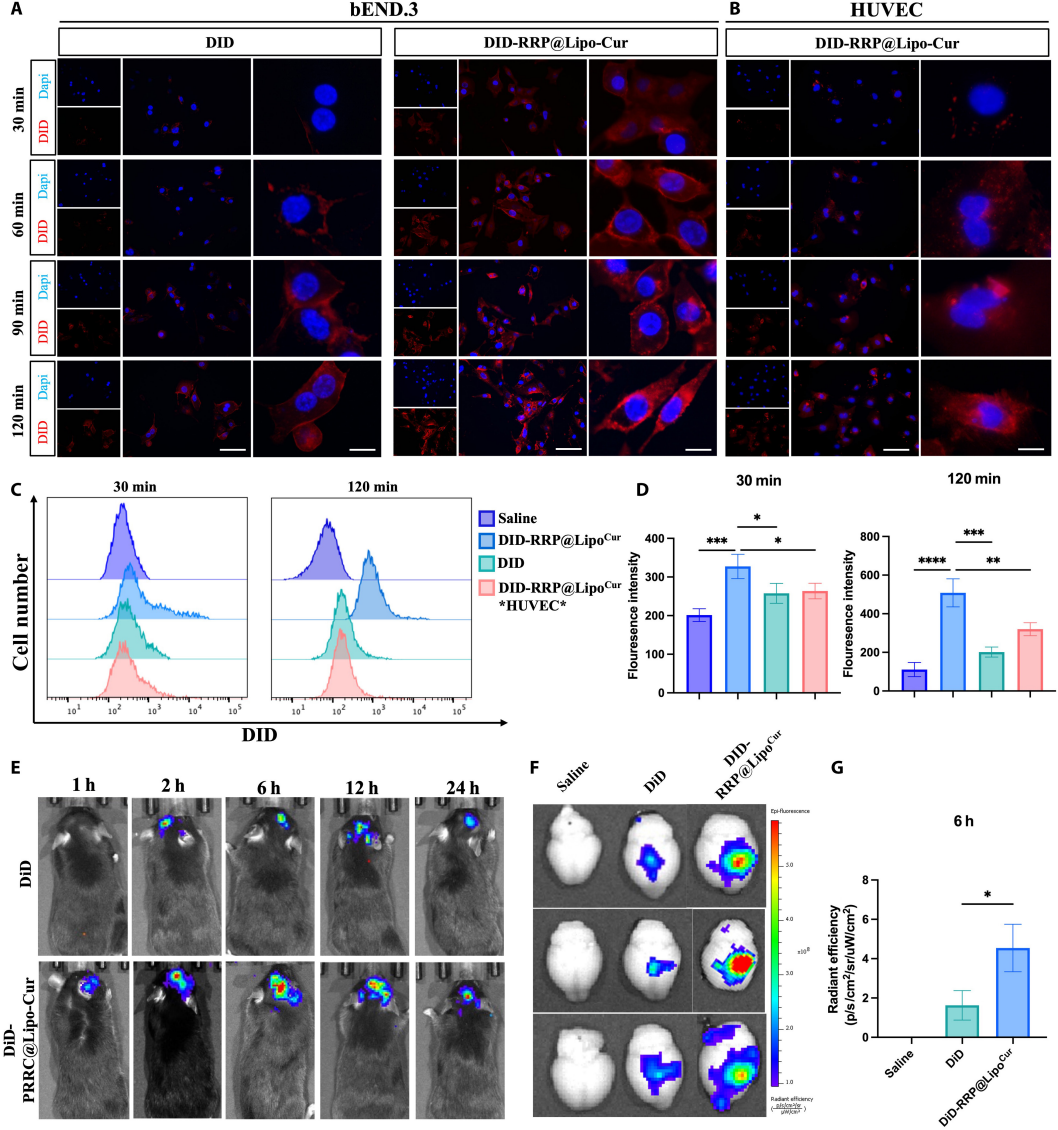
Biotargeting capability of RRP@Lipo-Cur both in vitro and in vivo. (A and B) Immunofluorescence was utilized to measure the distribution of DiD (red) in bEND.3 and HUVEC cultures treated with free DiD and DiD-RRP@Lipo-Cur at 30/60/90/120 min post-treatment. The nuclei were dyed with DAPI (blue). The scale bar from left to right is 100 μm and 10 μm. (C and D) Flow cytometry image of 4 groups (Saline group, DiD *bEND.3* group, DiD-RRP@Lipo-Cur *bEND.3* group, and DiD-RRP@Lipo-Cur *HUVEC* group) following respective treatments at 30- and 120-min post-treatment intervals, and the corresponding fluorescence intensity of each group was analyzed statistically (*n* = 3). *P < 0.05; **P < 0.01;***P < 0.001; ****P < 0.0001. (E) In vivo imaging system (IVIS) images of MCAO/r mice after injection of DiD-labeled RRP@Lipo-Cur and free DiD at 1, 2, 6, 12, and 24 h. (F and G) Ex vivo IVIS imaging of brains of 3 groups (Saline group, DiD group, and DiD-RRP@Lipo^Cur^ group) after 6 h of in vivo tracking. The radiant efficiency in brains from 3 groups at 6 h was calculated (*n* = 3). *P < 0.05.

1,1′-Dioctadecyl-3,3,3′,3′-tetramethylindodicarbocyanine (DiD) was employed as a marker in this study to identify the animal fluorescence localization and cell uptake of DiD-RRP@Lipo-Cur. As directly presented in Fig. 4A, addition of DiD-RRP@Lipo-Cur increased the fluorescence intensity of bEND.3 cells compared with addition of free DiD. From the fluorescence evidence at 4 time points, it can be concluded that DiD-RRP@Lipo-Cur is more rapidly absorbed by bEND.3 and remains high concentration within cells for a longer duration. Next, we used the peripheral vascular endothelial cell line HUVEC (human umbilical cord endothelial cell) and the central vascular endothelial cell line bEND.3 to verify the bioaffinity of DiD-RRP@Lipo-Cur for the CNS (Fig. 4B). Compared with bEND.3 cells, HUVEC internalized less DiD-RRP@Lipo-Cur showing lower fluorescence intensity at 4 time points, which indicated a higher CNS affinity of RRP@Lipo-Cur. We utilized flow cytometry to assess the variations in fluorescence intensity of the cells in each group after 30 and 120 min of coculture to further quantify the fluorescence intensity of the cells (Fig. 4C and D). The results were consistent with cell fluorescence. At 30 and 120 min, RRP@Lipo-Cur increased the uptake of DiD in bEND.3; RRP@Lipo-Cur showed higher affinity to bEND.3 cells.

Then, the biodistribution of RRP@Lipo-Cur in MCAO/r mice was investigated with the method of intravenous injection of free DiD or DiD-RRP@Lipo-Cur via the tail vein. The DiD-RRP@Lipo-Cur group exhibited significant accumulation in the ischemic brain at different intervals following administration in comparison to the free DiD, and considerable retention was still shown 24 h later, according to the real-time fluorescence pictures displayed in Fig. 4E. In addition, our findings revealed that the cumulative concentration of TLUS in the brain region reached its peak at 6 h post-injection. Consequently, we determined the intervention time point for TLUS to be at 6 h post-injection, thereby optimizing the efficacy of the synergistic treatment. Then, to further observe the accumulation of DiD in the brain, brains of mice were collected at 6 h after injection for live imaging. The results showed that the DiD-RRP@Lipo-Cur injection group had higher fluorescence aggregation and mainly concentrated in the ischemic hemisphere, which may be associated to the function of ROS-response peptide (Fig. 4F and G). Fluorescence imaging of brain section also proved this conclusion from histological aspect (Fig. S9). Taken together, these findings suggested that RRP@Lipo-Cur showed high affinity to CNS and proved its targeting ability. Finally, to validate the necessity of a nanotargeted carrier, we compared the protective efficacy of free curcumin + TLUS versus RRP@Lipo-Cur + TLUS against ischemia–reperfusion injury. The results from WB and TTC staining demonstrated that the RRP@Lipo-Cur + TLUS group exhibited superior neuroprotective effects under the same dosage of curcumin administration (Fig. S8A to F).

### RRP@Lipo-Cur + TLUS promoted motor functional recovery in CIRI mice

**Fig. 5.**
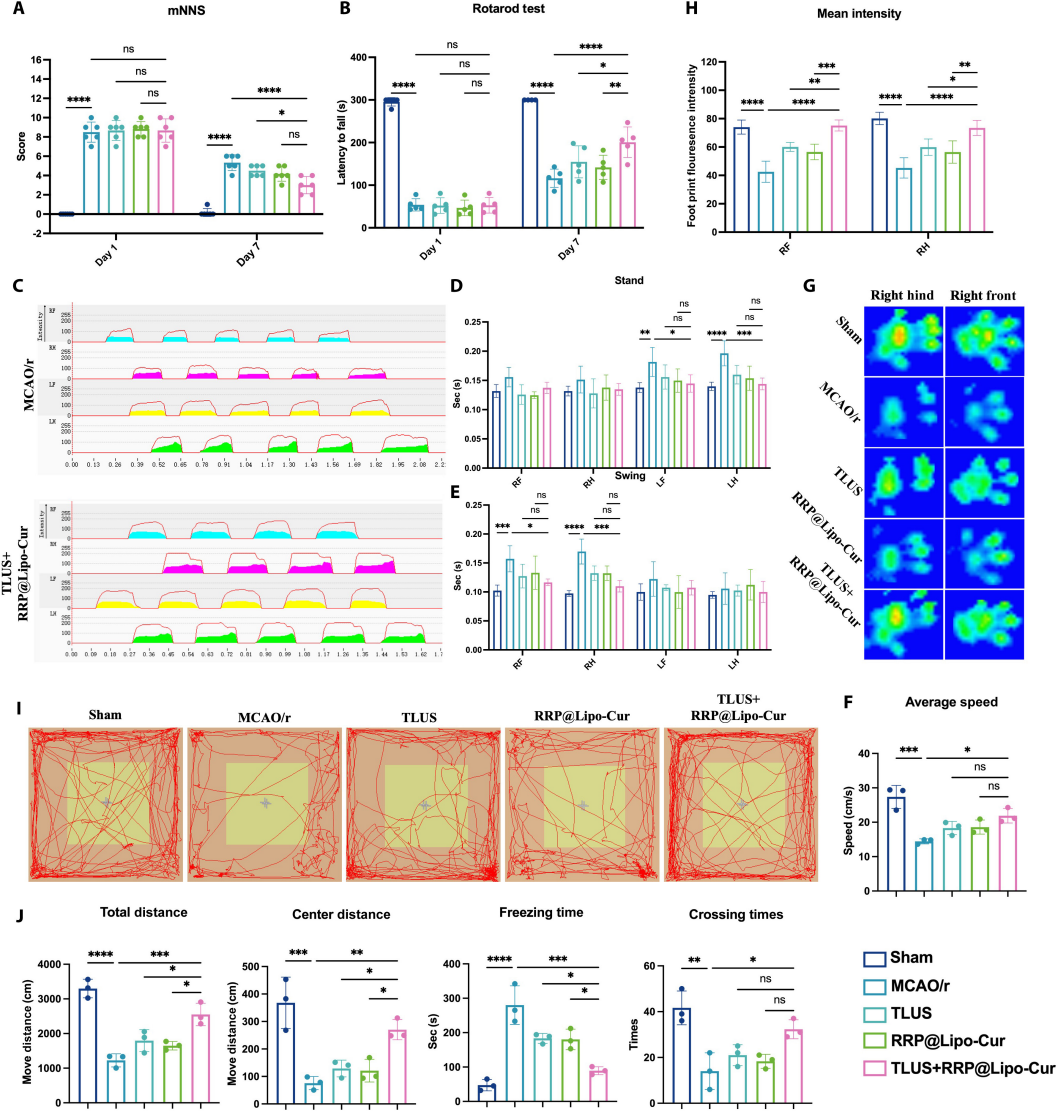
RRP@Lipo-Cur + TLUS promoted motor functional recovery in CIRI mice. (A and B) Statistical analysis of the score of mNSS and the duration time on the rotating bar in the rotarod test among 5 groups (Sham group, MCAO/r group, TLUS group, RRP@Lipo-Cur group, and TLUS + RRP@Lipo-Cur group) at days 1 and 7 (*n* = 6). ns, *P* > 0.05; *P < 0.05; **P < 0.01; ****P < 0.0001. (C to H) The CatWalk gait analysis system was employed to comprehensively compare locomotor ability and gait parameters across different experimental groups. The 2D gait pattern of MCAO/r group and TLUS + RRP@Lipo-Cur group. The comparison of stand time and swing time of 4 legs between the 5 groups (*n* = 4). ns; *P < 0.05; **P < 0.01; ***P < 0.001; ****P < 0.0001. The average speed of the mice in the different groups was compared (*n* = 4). ns, *P* > 0.05; *P < 0.05; ***P < 0.001. The footprint images of right hind and right front of mice, and the statistical analysis of paw mean intensity of right limbs in the 5 groups in the CatWalk gait test (*n* = 4). *P < 0.05; **P < 0.01; ***P < 0.001; ****P < 0.0001. (I and J) Representative images of motion trajectory diagrams of mice of different groups in the open-field test. Quantitative analysis of 4 motion parameters, including total distance, center distance, freezing time, and crossing times (*n* = 4). ns, *P* > 0.05; *P < 0.05; **P < 0.01; ***P < 0.001; ****P < 0.0001.

In this section of experiment, multiple animal behavioral tests were applied to verify the therapeutic effect of the synergistic therapy strategy on neurological function. In modified neurological severity score (mNSS) and rotarod test, no statistically significant difference was observed between MCAO/r and other 3 therapy groups at day 1, indicating that all mice were at the same motor function baseline after MCAO/r surgery. However, mice in the RRP@Lipo-Cur + TLUS group exhibited a better mNSS recovery and longer latency on rod compared to the MCAO/r group, RRP@Lipo-Cur group, and TLUS group at day 7 (Fig. 5A and B). Then, CatWalk gait analysis was used to detect recovery of gait control of mice in different groups. Mice receiving RRP@Lipo-Cur and TLUS combinative therapy presented a better gait control in stand of left limbs and swing of right limbs compared to mice in the MCAO/r group, TLUS group, and RRP@Lipo-Cur group (Fig. 5D and E). Besides, paw mean intensity of right limbs also showed better recovery after RRP@Lipo-Cur and TLUS synergistic therapy (Fig. 5G and H). We also measured the walking speed of the tested mice. The results demonstrated that the RRP@Lipo-Cur and TLUS combinative therapy significantly enhanced the walking speed of MCAO/r mice (Fig. 5F).

The 2-dimensional (2D) gait pattern of mice in the MCAO/r group and TLUS + RRP@Lipo-Cur group is provided in Fig. 5C, showing a clear gait recovery of mice after synergistic therapy. Next, the data analysis of the improved spontaneous exploratory activity assessed by the open field test (OPT) provided additional support for behavioral improvements after TLUS + RRP@Lipo-Cur treatment (Fig. 5I). Mice in the TLUS + RRP@Lipo-Cur group showed significant improvement in total distance, freezing time, center distance, and crossing times compared with mice in the MCAO/r group, TLUS group, and RRP@Lipo-Cur group (Fig. 5J). Taken together, these findings led us to a conclusion that combinative therapy strategy significantly ameliorated ischemic stroke-induced motor behavioral impairment and gait deficits. Notably, compared with single ultrasound or nanomaterial therapy, combinative therapy shows greater therapeutic potential on CIRI.

### RRP@Lipo-Cur + TLUS protected against CIRI-induced neuronal damage

**Fig. 6.**
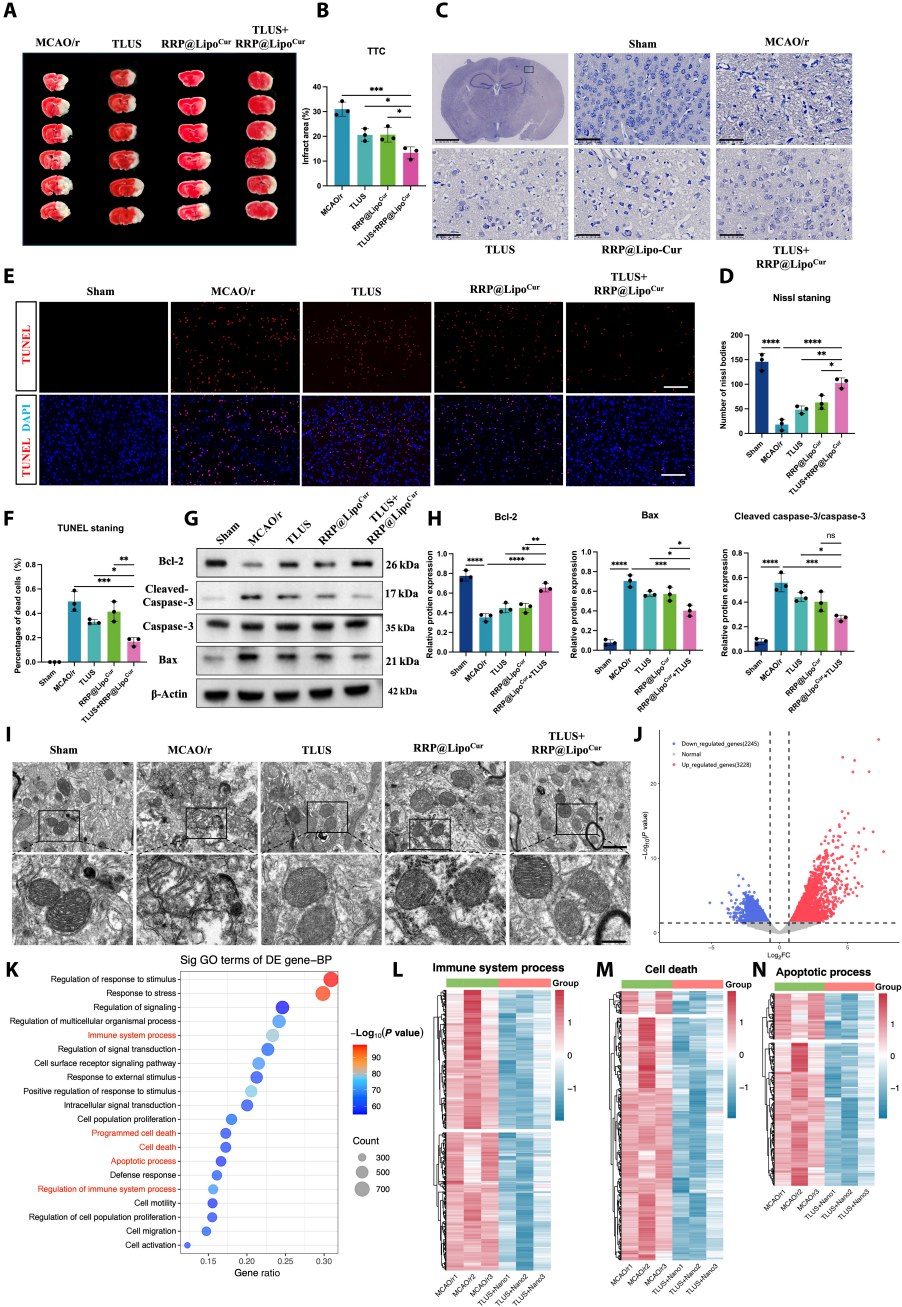
RRP@Lipo-Cur + TLUS protected against CIRI-induced neuronal damage. (A and B) TTC staining comparing effects of different groups (MCAO/r group, TLUS group, RRP@Lipo-Cur group, and TLUS + RRP@Lipo-Cur group) on cerebral infarction (white, infarcted regions; red, normal tissue). The infarct areas across groups were calculated (*n* = 3). *P < 0.05; ***P < 0.001. (C and D) Comparative analysis of Nissl staining was performed in the identical ischemic penumbra regions across 5 experimental groups. Scale bars, 1.25 mm and 50 μm. Quantitative assessment of the number of Nissl bodies was performed across all experimental groups (*n* = 3). *P < 0.05; **P < 0.01; ****P < 0.0001. (E and F) Immunofluorescence staining of TUNEL (red) was used to detect the relative expression and the distribution of dead cell in ischemic penumbra treated across the 5 groups. The nuclei were dyed with DAPI (blue). Scale bar, 100 μm. The percentage of dead cells was counted by calculating the ration of TUNEL and DAPI double-stained cells to the total number of DAPI-stained cells (*n* = 3). *P < 0.05; **P < 0.01; ***P < 0.001. (G and H) The protein expression of Bcl-2, cleaved caspase-3/caspase-3, and Bax in ischemic penumbra tissue across 5 groups was analyzed by WB (*n* = 3). *P < 0.05; **P < 0.01; ***P < 0.001; ****P < 0.0001. (I) TEM image of ischemic penumbra area in the 5 groups 7 d post-treatment (scale bars, 1 μm and 200 nm). (J) Volcano plot of DEGs between TLUS + RRP@Lipo-Cur and control groups in MCAO/r mouse brains. LogFC ≥ 1.5 and *P* < 0.05 were identified as significant changed DEGs. The red dots denote up-regulated genes, while the blue dots denote down-regulated genes. (K) Bubble plot visualization of GO annotation analysis for decreased DEGs was performed to explore the most biologically relevant processes associated with RRP@Lipo-Cur + TLUS therapeutic intervention. (L to N) Heatmap of gene expressions associated with “immune system processes”, “cell death”, and “apoptotic process” across 2 groups with 3 samples per group.

After confirming that RRP@Lipo-Cur + TLUS synergistic mode could considerably enhance the neurological function of MCAO/r mice, we further explored the protective effect of this treatment mode on neurons in the ischemic penumbra. First, TTC staining showed that, in comparison to the MCAO/r group and the other 2 single treatment groups, RRP@Lipo-Cur + TLUS significantly reduced the infarct size (Fig. 6A and B). Then, Nissl staining for Nissl bodies and terminal deoxynucleotidyl transferase–mediated deoxyuridine triphosphate nick end labeling (TUNEL) staining for apoptosis cells in the ischemic periphery showed that the RRP@Lipo-Cur + TLUS therapy significantly increased the number of survival cells and ameliorated the number of cell death. Meanwhile, the therapeutic effects were significantly better than in other 2 treatment groups (Fig. 6C to F). Subsequently, tissues from ischemic penumbra area of 5 groups were obtained to detect the expression of apoptosis-related proteins by WB, and the results further verified the fact that the RRP@Lipo-Cur + TLUS therapy strategy exhibited greater neuroprotective effect (Fig. 6G and H). TEM was also applied to observe ischemic penumbra area. After cerebral ischemia–reperfusion, ultrastructural imaging revealed notable alterations in mitochondrial morphology, such as swelling, cristae breakage, and a decrease in the number of cristae. However, RRP@Lipo-Cur + TLUS treatment strategy reversed these morphology changes of mitochondria and showed better therapeutic effect than single intervention strategies (Fig. 6I). According to the results presented above, treatment with RRP@Lipo-Cur + TLUS demonstrated more superior efficacy in correcting CIRI-induced neuronal damage. Last, transcriptome-wide RNA sequencing technique, accompanied by subsequent bioinformatics analysis, was conducted to investigate the potential mechanism by which RRP@Lipo-Cur + TLUS mitigates neuronal damage caused by cerebral ischemia. We found 5,673 differentially expressed genes (DEGs), with 3,228 genes up-regulated and 2,445 genes down-regulated between MCAO/r and RRP@Lipo-Cur + TLUS groups (logFC ≥ 1.5 and *P* < 0.05) (Fig. 6I and Fig. S10A). Raw DEG table is provided in Table S3. We additionally utilized Gene Ontology (GO) annotation analysis of down-regulated DEGs to determine the most related biological functions induced by RRP@Lipo-Cur + TLUS treatment. Among the top 20 enriched biological functions, the biological processes of “immune system process”, “cell death”, “programmed cell death”, “apoptotic process”, and “regulation of immune system process” indicated that RRP@Lipo-Cur + TLUS synergistic therapy probably protected ischemic stroke via an anti-inflammatory and anti-apoptotic mechanism (Fig. 6K). Besides, the heatmaps of DEGs included in these 5 biological processes were also presented (Fig. 6L to N and Fig. S10B and C). Based on the results of the aforementioned analysis, we further conducted ​gene set enrichment analysis (GSEA) focusing on neuronal inflammation and neuronal apoptosis. The findings also revealed a significant reduction in neuroinflammation and neuronal apoptosis following synergistic therapy (Fig. S10D and E). In the subsequent study, we conducted in vitro experiments to independently investigate the therapeutic impact of synergistic treatment on microglial polarization and its anti-apoptotic effect on neurons, aiming to validate the findings obtained from RNA sequencing.

### RRP@Lipo-Cur + TLUS suppressed OGD/r-induced inflammation via mediating microglia polarization

**Fig. 7.**
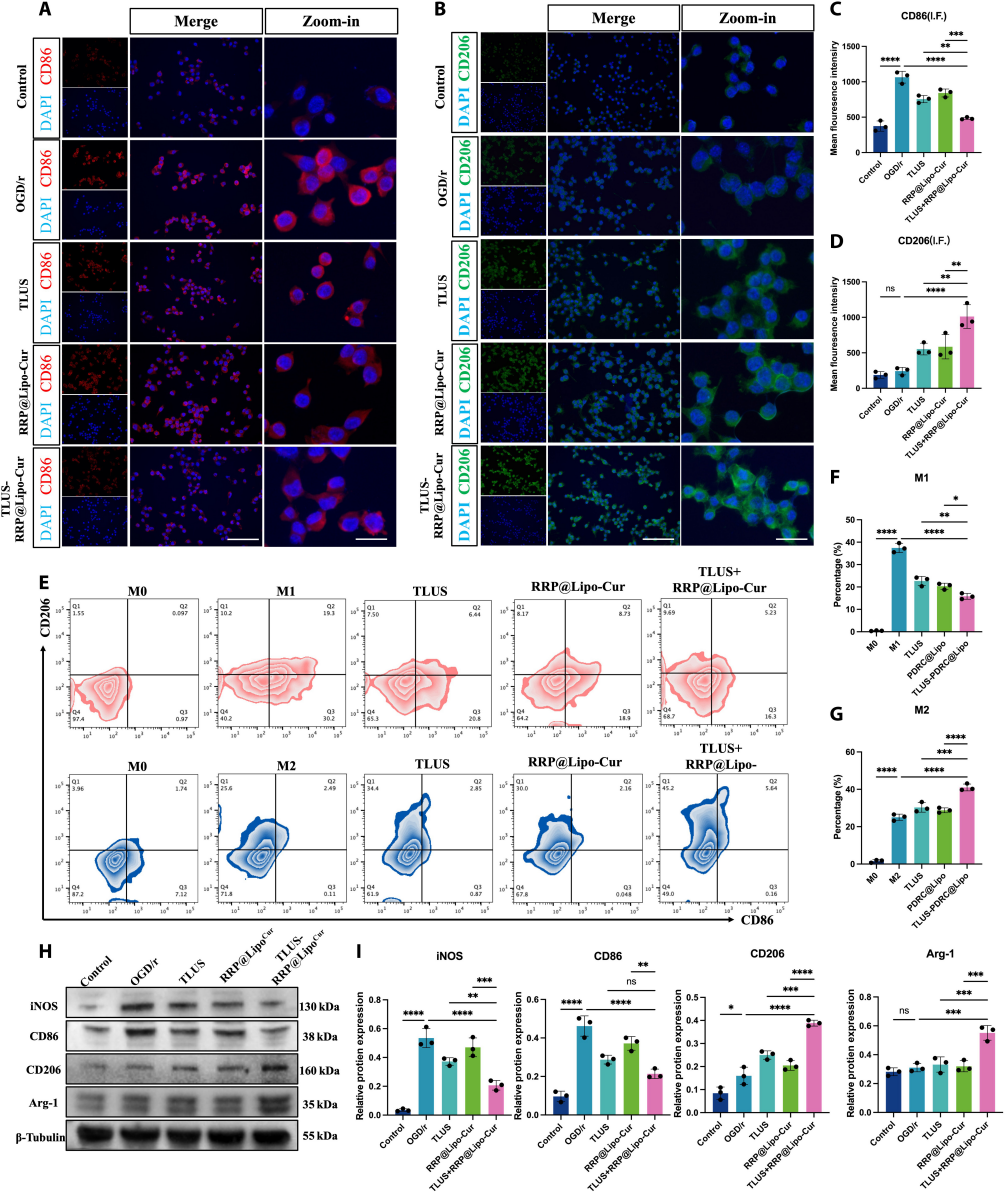
RRP@Lipo-Cur + TLUS suppressed OGD/r-induced inflammation via mediating microglia polarization. (A) Representative immunofluorescence image of CD86, marker of M1 BV-2 cells, among 5 groups. (B) The mean fluorescence intensity of CD86 was calculated by dividing the total red fluorescence intensity by the number of DAPI-stained nuclei. Then, the mean fluorescence intensity among 5 groups was statistically analyzed (*n* = 3). **P < 0.01; ***P < 0.001; ****P < 0.0001. (C) Representative immunofluorescence image of CD206, marker of M2 BV-2 cells, among 5 groups. (D) The mean fluorescence intensity of CD206 was calculated by dividing the total green fluorescence intensity by the number of DAPI-stained nuclei. Then, the mean fluorescence intensity among 5 groups was statistically analyzed (*n* = 3). **P < 0.01; ****P < 0.0001. (E) Representative flow cytometry profile of CD86- and CD206-positive cells. (F) Statistical chart of cell distribution in each group after receiving different treatments under M1 model. We defined the lower right quadrant as the proportion of M1-positive cells. (G) Statistical chart of cell distribution in each group after receiving different treatments under M2 model (*n* = 3). *P < 0.05; **P < 0.01; ****P < 0.0001. We defined the upper left quadrant as the proportion of M1-positive cells (*n* = 3). ***P < 0.001; ****P < 0.0001. (H) The expression levels of iNOS, CD86, CD206, and Arg-1 in BV-2 cells across 5 groups were determined by WB (*n* = 3). (I) Statistical chart of iNOS, CD86, CD206, and Arg-1. *P < 0.05; **P < 0.01; ***P < 0.001; ****P < 0.0001.

To further test the findings obtained from RNA sequencing, that is, RRP@Lipo-Cur + TLUS protects the neurons in the ischemic penumbra mainly through inhibiting neuroinflammatory and neuron apoptotic processes, we used oxygen and glucose deprivation reperfusion (OGD/r)-induced HT-22 cells and OGD/r-induced BV-2 cells to simulate the inflammatory and apoptotic processes occurring after ischemia–reperfusion in vivo. However, as WB and apoptosis flow cytometry demonstrated in Fig. S11A to D, RRP@Lipo-Cur + TLUS failed to significantly ameliorate OGD/r-induced cell apoptosis. Therefore, rather than a direct therapeutic effect on hypoxic–ischemic neurons, we hypothesized that the neuroprotective effect of RRP@Lipo-Cur + TLUS on hypoxic–ischemic neurons was probably achieved by inhibiting the excessive inflammatory response in the ischemic peripheral region. As the principal source of neuroinflammatory cytokines, microglia play a fundamentally critical role in neuroinflammation and are capable of modulating a wide array of cellular responses [29]. Besides, microglial polarization is the primary phenotypic manifestation of neuroinflammatory alterations [30]. Therefore, we conducted an analysis of microglial polarization to elucidate the primary neuroprotective mechanism underlying the synergistic treatment. First, we assessed the regulatory effect of RRP@Lipo-Cur + TLUS on OGD/r-induced BV-2 cells. Immunofluorescence of CD86 and CD206, which are marker proteins of M1 and M2 microglia, respectively, showed a significant improvement in polarization of BV-2 after RRP@Lipo-Cur + TLUS treatment, and RRP@Lipo-Cur + TLUS presented a superior regulatory effect compared to single TLUS or single RRP@Lipo-Cur + TLUS intervention (Fig. 7A to D). Then, flow cytometry was used to analyze microglia polarization states after different intervention strategy from cellular aspect. The cell distribution of M1 and M2 BV-2 indicated that RRP@Lipo-Cur + TLUS had more prominent effect on suppressing M1 polarization and enhancing M2 polarization, which was consistent with immunofluorescence (Fig. 7E to G). Last, WB was applied to confirm the relative protein expression of CD86, inducible nitric oxide synthase (iNOS), CD206, and Arg-1. The results showed that RRP@Lipo-Cur + TLUS treatment not only significantly suppressed the inhibited protein expression of CD86 and iNOS but also significantly promoted the protein expression of Arg-1 and CD206. Consistent with the results of immunofluorescence and flow cytometry, analysis of protein expression further indicated that the combinative treatment had a better regulation effect on microglia polarization than the single treatment (Fig. 7H and I). The results above led us to the conclusion that RRP@Lipo-Cur + TLUS could effectively regulate ischemia reperfusion-mediated neuroinflammation by promoting M2 microglia polarization and inhibiting M1 microglia polarization. Cytokines are the main bridge of communication between microglia and neurons, especially in the case of extensive inflammation. When M1 microglial cells are dominant, proinflammatory factors are widely released, which will produce an inflammatory storm and further intensify the damage of ischemic and hypoxic neurons. Meanwhile, the release of anti-inflammatory factors by M2 microglia is significantly reduced, and the protective effect on ischemic and hypoxic neurons is also weakened [31]. Therefore, we further investigated the intracellular and extracellular expression of proinflammatory cytokines and anti-inflammatory cytokines in OGD/r-induced BV-2 after different intervention. The enzyme-linked immunosorbent assay (ELISA) and WB (Fig. S12A to C) demonstrated consequently that RRP@Lipo-Cur + TLUS significantly inhibited the expression of proinflammatory cytokines [IL-1β and tumor necrosis factor-α (TNF-α)] but significantly promoted anti-inflammatory cytokines [transforming growth factor-β (TGF-β), interleukin-4 (IL-4), and IL-10]. Based on this, including the modulatory effect on microglia polarization and inflammatory cytokines but no direct anti-apoptotic effect on neurons, we hypothesized that RRP@Lipo-Cur + TLUS protect hypoxic–ischemic neurons by modulating the expression of pro- and anti-inflammatory cytokines by influencing microglial polarization.

### RRP@Lipo-Cur + TLUS attenuates OGD/r-modulated neuronal apoptosis by balancing microglia-derived cytokines

**Fig. 8.**
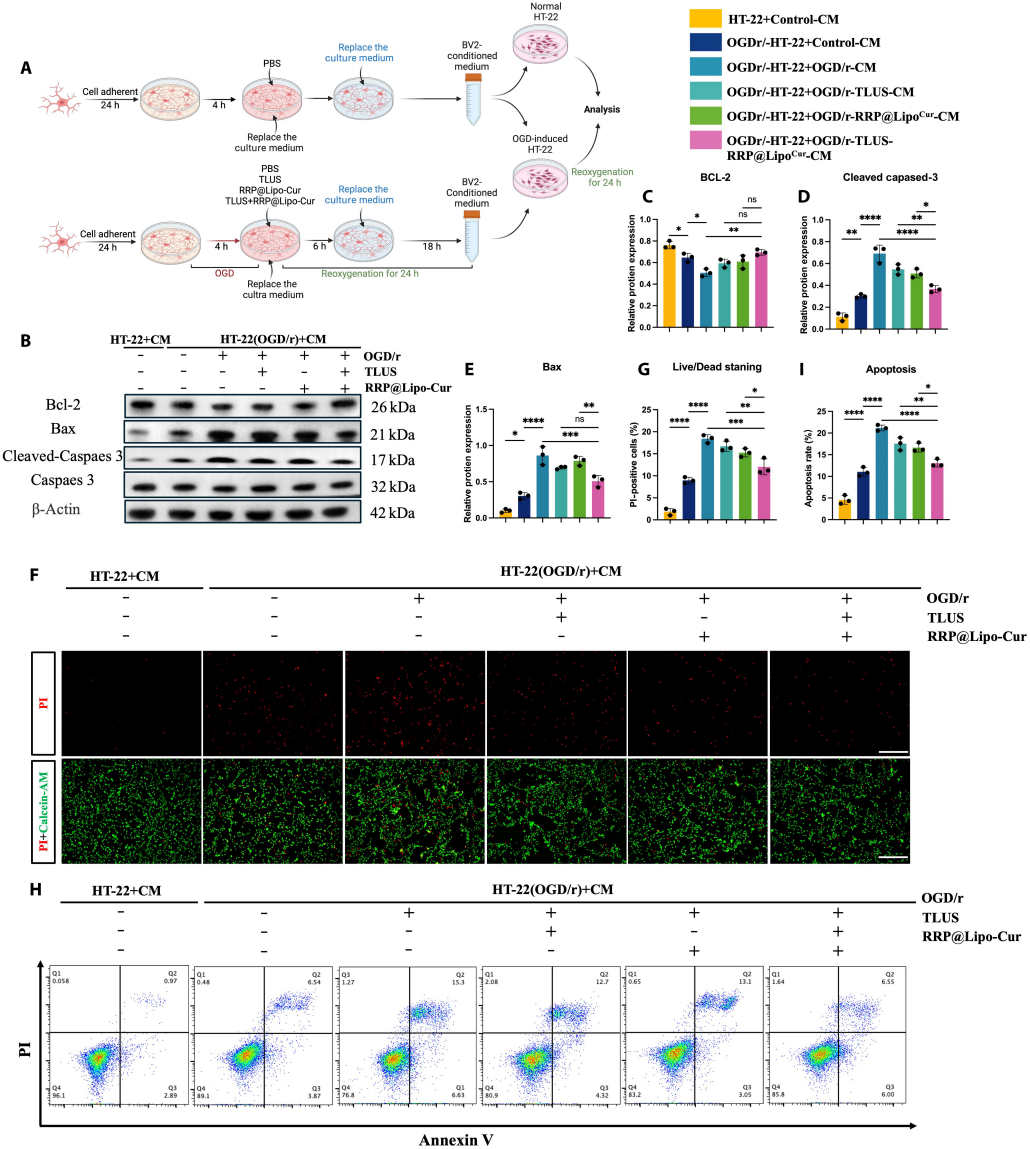
RRP@Lipo-Cur + TLUS attenuates OGD/r-modulated neuronal apoptosis by balancing microglia-derived cytokines. (A) Schematic workflow of BV-2 microglial CM experimental design. (B to E) Changes in protein expression of Bcl-2, cleaved caspase-3/caspase-3, and Bax in the 6 groups (HT-22 + Control-CM group, OGD/r-HT-22 + Control-CM group, OGD/r-HT-22 + OGD/r-CM group, OGD/r-HT-22 + OGD/r-TLUS-CM group, OGD/r-HT-22 + OGD/r-RRP@LipoCur-CM group, and OGD/r-HT-22 + OGD/r-TLUS-RRP@LipoCur-CM group) were recorded and analyzed by WB (*n* = 3). ns, *P* > 0.05; *P < 0.05; **P < 0.01; ***P < 0.001; ****P < 0.0001. (F and G) Live/Dead staining was used to observe the number and percentage of dead cells (PI: red) and live cells (calcein AM: green) in 6 groups of HT-22 cells (*n* = 3). *P < 0.05; **P < 0.01; ***P < 0.001; ****P < 0.0001. (H and I) Flow cytometry analysis of cell death and intergroup apoptosis rate comparison in PI-stained HT-22 cells across 6 conditions (*n* = 3). *P < 0.05; **P < 0.01; ****P < 0.0001.

Although the modulatory effect of synergistic treatment on microglial polarization has preliminarily been elucidated, it remains unclear how it further confers indirect protective effect on neuron. In this part of study, to further elucidate whether RRP@Lipo-Cur + TLUS protect neurons from ischemia–reperfusion injury by balancing microglia-derived cytokines, we applied conditioned medium (CM) from BV-2 cell after different treatments to cultivate OGD/r-induced HT-22 cells. In previous part of the research, we found a favorable modulation in the levels of microglia-derived proinflammatory and anti-inflammatory cytokines with RRP@Lipo-Cur + TLUS treatment. Based on this, expression of apoptosis-related protein Bax, cleaved caspase-3/caspase-3, and Bcl-2 was further detected to determine the apoptosis levels of OGD/r HT-22 cells by WB. Compared with incubation with CM collected from the nontreated, TLUS-treated, and RRP@Lipo-Cur-treated OGD/r BV-2, CM collected from OGD/r BV-2 treated with collaborative therapy of RRP@Lipo-Cur + TLUS significantly inhibited expression of Bax and cleaved caspase-3 but promoted the expression of anti-apoptotic protein Bcl-2 (Fig. 8B to E). In addition, Live/Dead and apoptosis flow cytometry was also applied to further evaluate the apoptosis rate of HT-22 cells. Both tests illustrated that CM collected from RRP@Lipo-Cur + TLUS-treated BV-2 significantly alleviated secondary cell apoptosis of neurons (Fig. 8F to I). Compared to the effect of single RRP@Lipo-Cur treatment and single TLUS treatment on BV-2, combinative strategy showed superior modulating effects on microglia-secreted extracellular environment. In conclusion, it can be preliminarily concluded that the synergistic treatment directly modulates microglia polarization but exerts indirectly neuroprotective effects on neurons through the regulation of microglial polarization and the associated inflammatory cytokines.

### RRP@Lipo-Cur + TLUS modulated MCAO/r-induced neuroinflammatory microenvironment by regulating microglia polarization

**Fig. 9.**
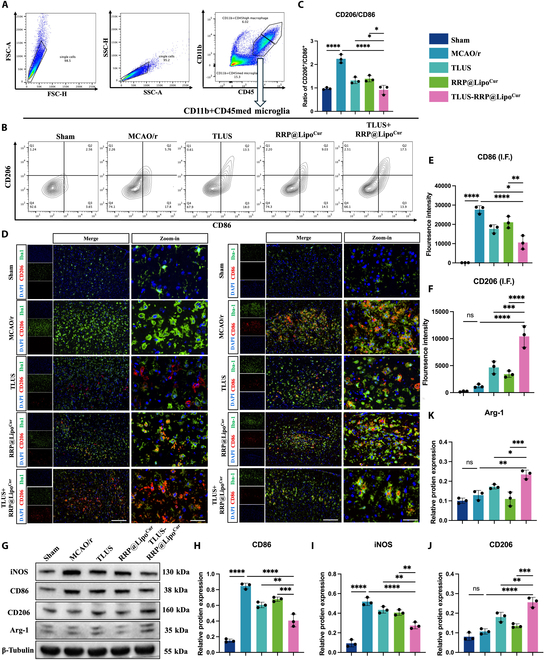
RRP@Lipo-Cur + TLUS modulated MCAO/r-induced neuroinflammatory microenvironment by regulating microglia polarization. (A to C) Comprehensive cellular characterization by flow cytometry with microglia identification through CD45/CD11b coexpression analysis, and comparative analysis of CD86- and CD206-positive microglia in population of CD11^+^CD45^+^med microglia among 5 treatment groups (Sham group, MCAO/r group, TLUS group, RRP@Lipo-Cur group, and TLUS + RRP@Lipo-Cur group). The upper right quadrant was defined as M2 cells, and the lower right quadrant was defined as M1 cells. The ratio of M2 cells/M1 cells was calculated (*n* = 4). *P < 0.05; ****P < 0.0001. (D to F) Immunofluorescence was employed to measure the relative expression level and distribution of CD86 (green) and CD206 (red) among the 5 groups. The nuclei were dyed with DAPI (blue). The scale bars from left to right is 500 μm and 100 μm. The fluorescence intensity of CD86- and CD206-positive cells was counted by the ratio of the area of red fluorescence to the total area (*n* = 3). ns *P* > 0.05; *P < 0.05; **P < 0.01; ***P < 0.001; ****P < 0.0001. (G to K) The protein expression of CD86, iNOS, CD206, and Arg-1 across 5 groups was determined and statistically analyzed by WB (*n* = 3). ns, *P* > 0.05; *P < 0.05; **P < 0.01; ***P < 0.001; ****P < 0.0001.

Next, we further investigated whether this therapeutic approach exerts neuroprotection through the same biological processes in ischemic penumbra of MCAO/r mice. As shown in brain ischemic penumbra flow cytometry, CD11b^+^CD45^+med^ [32] and CD11b^+^CD45^+high^ were applied to label microglia and macrophages, respectively [33] (Fig. 9A). Then, CD11b^+^CD45^+med^-labeled microglia were further analyzed via CD86 and CD206, markers of the M1 and M2 phenotype microglia, respectively. Among 5 groups, TLUS + RRP@Lipo-Cur synergistic therapy significantly regulated the proportion of microglia polarization, which was consistent with in vitro results (Fig. 9B). Compared with single treatment, synergistic treatment showed a better therapeutic effect (Fig. 9C). Then, immunofluorescence (Fig. 9D to F) and immunoblot (Fig. 9G to K) were applied to further confirm effect of synergistic treatment on microglia. Both experiments proved that the polarization balance of microglia was significantly improved after treatment of synergistic combination of TLUS and TLUS + RRP@Lipo-Cur. Meanwhile, compared to single TLUS or single TLUS + RRP@Lipo-Cur treatment, synergistic therapy also significantly increased the expression of CD206 and Arg-1 but decreased the expression of CD86 and iNOS, showing a superior effect. Meanwhile, the pro- and anti-inflammatory cytokines in ischemic penumbra were also detected with ELISA (Fig. S13). The results also showed that the neuroinflammatory cytokines in the ischemic penumbra were effectively regulated after the combined treatment, suggesting that the combined treatment optimized the inflammatory microenvironment around the neurons. The aforementioned results were consistent with the findings in vitro, further elucidating the potential biological mechanisms underlying the synergistic treatment.

### Depletion of microglia in MCAO/r mice inhibited the neuroprotective effect of RRP@Lipo-Cur + TLUS

**Fig. 10.**
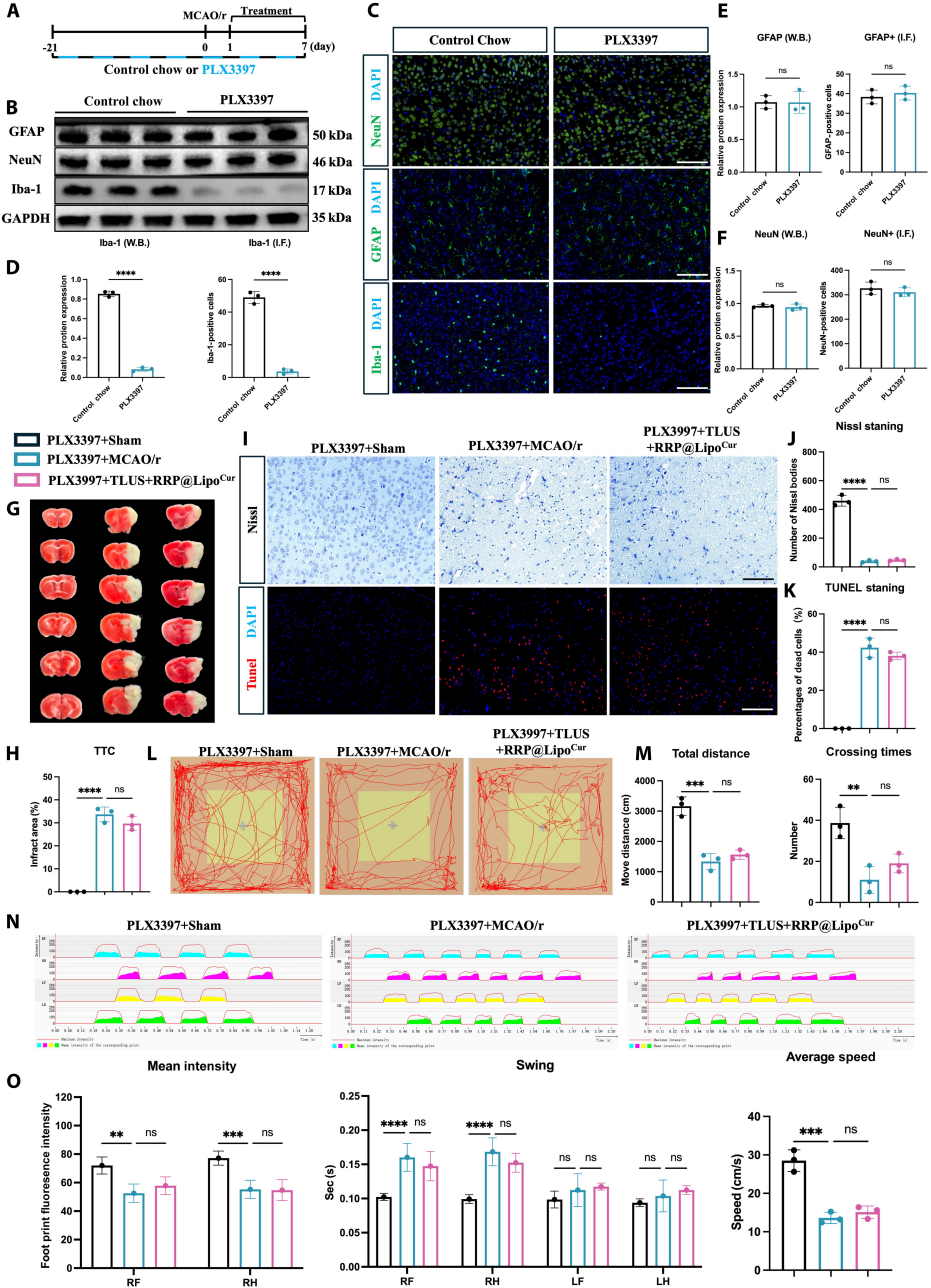
Depletion of microglia in MCAO/r mice inhibited the neuroprotective effect of RRP@Lipo-Cur + TLUS. (A) Experimental timeline for establishing microglia-depleted MCAO/r mouse model with PLX3397 dietary regimen. (B to F) WB and immunofluorescence analysis of 3 target proteins (green), including GFAP, NeuN, and Iba-1, in mice fed control or PLX3397-containing diet. The nuclei were dyed with DAPI (blue). The expression for the 3 proteins was quantified by calculating the ratio of green and DAPI double-stained cells to the total number of DAPI, respectively (*n* = 3). ns, *P* > 0.05; ****P < 0.0001. (G and H) TTC staining comparing the effects of 3 groups (PLX3397 + Sham group, PLX3397 + MCAO/r group, and PLX3397 + TLUS + RRP@Lipo-Cur group) on cerebral infarction (white, infarcted regions; red, normal tissue). The infarct areas across groups were counted (*n* = 3). ns, *P* > 0.05; ****P < 0.0001. (I to K) Comparative analysis of Nissl and TUNEL staining (red, TUNEL; blue, DAPI) in the ischemic penumbra among the 3 groups. The number of Nissl bodies and percentage of dead cells were calculated (*n* = 3). ns, *P* > 0.05; ****P < 0.0001. (L and M) Representative images of motion trajectory diagrams of the 3 groups in the open-field test. Quantitative analysis of 2 motion parameters, including total distance and crossing times (*n* = 3). ns, *P* > 0.05; **P < 0.01; ***P < 0.001. (N and O) The CatWalk gait analysis system was used to compare gait parameters across 3 groups. The 2D gait pattern of PLX3397 + Sham group, PLX3397 + MCAO/r group, and PLX3397 + TLUS + RRP@Lipo-Cur group. The comparison of mean intensity of right limbs, swing time of 4 legs, and average speed between the 3 groups (*n* = 4). ns, *P* > 0.05; **P < 0.01; ***P < 0.001; ****P < 0.0001.

To further confirm that microglia are the key effector cells involved in the synergistic treatment, we employed a mouse model featuring microglial depletion. Microglial survival depends on the colony-stimulating factor 1 receptor (CSF1R) receptor [34]. Thus, PLX3397, a CSF1R inhibitor providing a noninvasive way to reduce microglia without causing brain damage or cognitive/behavioral abnormalities, was applied [35]. Figure 10A illustrates the timeline of establishment of microglial depletion. Immunoblot and immunofluorescence showed that 21-d continuous PLX3397 feeding significantly reduced the Iba-1-positive microglia and protein expression of Iba-1 (Fig. 10D). Besides, no significant changes were observed in immunofluorescence and protein expression of glial fibrillary acidic protein (GFAP) and neuronal nuclear (NeuN), which indicated that PLX3397 treatment had no effect on neurons and astrocyte (Fig. 10E and F). Meanwhile, the OPT and CatWalk gait analysis were applied 1 d before MCAO/r surgery. The results showed no significant difference between the control chow group and the PLX3397 group (Fig. S14A to C). The data above indicated the successful establishment of microglial depletion and unaffected behavioral function. Then, the therapeutic effect of RRP@Lipo-Cur + TLUS was detected under MCAO/r and microglial depletion background. No significant difference was observed in changes of infarct size in TTC staining after RRP@Lipo-Cur + TLUS treatment (Fig. 10G and H). The number of Nissl bodies and apoptotic cells in the ischemic penumbra did not significantly change after synergistic therapy (Fig. 10I to K). Last, the results from neurobehavioral experiments showed that RRP@Lipo-Cur + TLUS therapy failed to ameliorate the CIRI-induced kinetic parameters [swing time, average speed, and mean intensity (Fig. 10O)]. Similarly, no statistically significant differences were observed between PLX3397 + MCAO/r and RRP@Lipo-Cur + TLUS + PLX3397 + MCAO/r groups in total distance and crossing time (Fig. 10M). In conclusion, the results above revealed that microglia depletion reversed the therapeutic effect of synergistic treatment and suggested that synergistic treatment primarily exerts its effects through the modulation of microglia.

### RRP@Lipo-Cur + TLUS exert its neuroprotective effect through NF-κB and MAPK signaling pathway in microglia

**Fig. 11.**
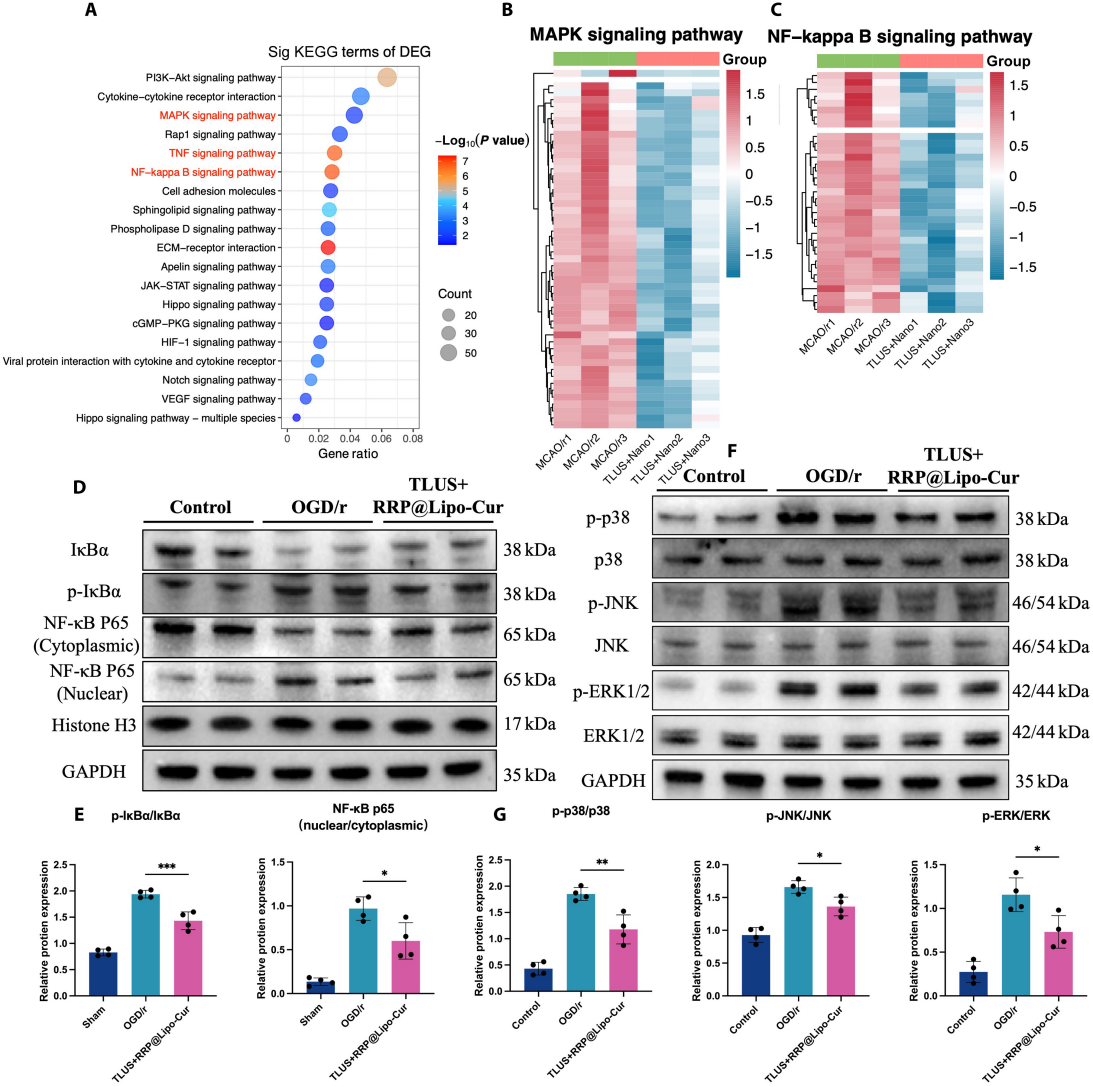
RRP@Lipo-Cur + TLUS exert its neuroprotective effect through NF-κB and MAPK signaling pathway in microglia. (A) Bubble plot of top 20 pathways after KEGG analysis for decreased DEGs between the RRP@Lipo-Cur + TLUS group and the MCAO/r group. (B and C) Heatmap of gene expressions associated with MAPK signaling pathway and NF-κB signaling pathways across 2 groups with 3 samples per group. (D to G) WB analysis was performed to evaluate the expression of key proteins in the MAPK and NF-κB signaling pathways, including IκBα, p-IκBα, NF-κB P65 (cytoplasmic), NF-κB P65 (nuclear), histone H3, p-p38, p38, p-JNK (c-Jun N-terminal kinase), JNK, p-ERK1/2 (extracellular signal–regulated kinase 1/2), and ERK1/2, across 3 groups (Control group, OGD/r group, and TLUS + RRP@Lipo-Cur group). The relative expression of these proteins was quantified (*n* = 4). *P < 0.05; **P < 0.01; ***P < 0.001.

Subsequently, we utilized in vitro BV-2 cells to further investigate the intracellular mechanisms underlying the regulatory effects of synergistic treatment on microglia polarization. We employed Kyoto Encyclopedia of Genes and Genomes (KEGG) enrichment analysis. The most significantly enriched inflammation-related pathways included the “MAPK signaling pathway” and the “NF-kappa B (NF-κB) signaling pathway” (Fig. 11A). The heat maps of significantly down-regulated genes in both pathways are also shown in Fig. 11B and C. To further elucidate the mechanisms potentially involved in the modulation of microglial phenotypes following synergistic therapy, we subjected BV-2 cells to OGD/r and found that both pathways were significantly up-regulated in the post-OGD/r treatment. However, intervention with synergistic treatment significantly attenuated the activation of the MAPK signaling pathway and the nuclear translocation of NF-κB with WB analysis.

In addition, KEGG enrichment analysis revealed that the TNF signaling pathway was significantly suppressed following treatment, further corroborating the regulatory impact of the combined treatment on proinflammatory cytokines (Fig. 11A and Fig. S15A to D). These findings suggested that the MAPK/NF-κB signaling pathway served as a critical participant in modulating microglial polarization and regulating the inflammatory microenvironment surrounding neurons (Fig. 11D to G).

### Safety of RRP@Lipo-Cur

We verified the safety of RRP@Lipo-Cur by detecting blood of mice and H&E staining of various organs. Study on blood biochemical indicators showed that no significant changes on aspartate aminotransferase (AST), alanine aminotransferase (ALT) (Fig. S16A), blood urea nitrogen (BUN), creatinine (CREA), and uric acid (UA) (Fig. S16B) were found, suggesting that there was no discernible hepatic or renal damage with RRP@Lipo-Cur injection. At last, the H&E sections from major tissue of different treated mice showed that the RRP@Lipo-Cur group did not exhibit any noticeable physiological changes (Fig. S16C). All these results, taken together, demonstrated the biocompatibility of RRP@Lipo-Cur in vivo settings.

## Discussion

Currently, the cornerstone principle in the clinical management of ischemic stroke is to achieve timely restoration of blood reperfusion of the ischemic penumbra within the time window, thereby ensuring that brain tissue can adequately replenish oxygen and salvage compromised neurons [36]. However, reperfusion will still exacerbate neural damage, including metabolic alterations, ROS overexpression, dysregulation of autophagy, and mitochondrial dysfunction, if the treatment is not timely intervened [37]. All these factors will result in not only further neuronal death but also an excessive inflammatory response within the ischemic penumbra [38]. Thus, safeguarding neurons and fine-tuning the inflammatory response within the ischemic penumbra are pivotal in facilitating the recovery from cerebral ischemia [39]. Owing to the low permeability of the BBB, insufficient cumulative concentration in the lesion area, and systemic side effects, traditional drug therapies are unable to fully realize their therapeutic potential [40]. Thus, we developed a dual stimuli-responsive neuron-targeted drug delivery liposome in this study. After being targeted to the brain via the RVG29 peptide, this smart liposome is capable of triggering the release of loaded curcumin in response to stimulation by ROS and TLUS. This indicates that it can first target the brain region and subsequently release curcumin [41] through both disease site specificity and artificially guided selection, thereby significantly enhancing treatment efficacy. Continuous ultrasound stimulation, in conjunction with the release of curcumin, will also exert a therapeutic effect in the cotreatment of ischemic stroke. In this study, we primarily proved that the in vitro release efficiency of curcumin was significantly increased under 10 μM H_2_O_2_ or 300 mW/cm^2^ ultrasound stimulation. Meanwhile, the RVG29 peptide effectively improved the affinity of nanocarrier to brain in vivo and brain-derived endothelial cell in vivo.

Through RNA sequencing, we discovered that neuroprotective benefits and behavioral improvement of synergistic therapy in acute ischemia–reperfusion mice were mainly due to its regulation of the biological processes of inflammation and cell death. As the primary resident immune cells in the brain, the substantial accumulation of polarized microglia in the penumbra represents a critical neuroinflammatory process following ischemic stroke, leading to neuronal loss [42]. Given the results of the cell investigations, we discovered that the synergistic treatment significantly regulated microglia polarization but had no discernible beneficial effect on hypoxic–ischemic neurons, which revealed the possibility that the intercellular communication between inhibited inflammation and neurons plays a major role on protecting neuronal loss. Our subsequent CM research proved our hypothesis and showed that RRP@Lipo-Cur + TLUS altered the composition of inflammatory cytokines in the supernatant of BV-2 cells and reduced neuronal death, which was also verified in the animal experiment. In the acute phase, M1-type microglia predominate during neuroinflammation and aggravate neuronal death by secreting TNF-α and other proinflammatory factors [43]. Anti-inflammatory cytokines produced by M2 microglia not only help to remodel damaged areas and promote angiogenesis but also inhibit cell apoptosis and promote neuronal growth and repair [44]. Therefore, the dynamic equilibrium between M1 and M2 phenotypes plays a crucial role in determining neuronal survival and the outcome of ischemic stroke [45].

Low-intensity pulsed ultrasound has been documented to modulate inflammatory responses in the management of various pathological conditions [46]. Hong et al. [47] found that TLUS could significantly alleviate NLRP3-related neuroinflammation induced by ischemic stroke. Besides, Wang et al. [48] demonstrated that transcranial focused ultrasound stimulation increased M2 microglia in the ischemic brain region. Beyond its neuroinflammatory modulatory effects, TLUS can transiently open the BBB, thereby potentiating the targeted delivery of therapeutic agents with enhanced efficacy [49]. Ultrasound stimulation has been demonstrated to significantly augment cell membrane permeability, thereby enhancing the exchange of substances between the intracellular and extracellular milieus [50]. Curcumin has also been widely reported to exert a profound regulatory influence on microglia-mediated neuroinflammation [51]. Ran et al. [52] demonstrated that intraperitoneal administration of curcumin protects against white matter injury following cerebral ischemia by inhibiting microglia/macrophage pyroptosis. Liu et al. [53] also elucidated that intraperitoneal administration of curcumin at a dose of 150 mg/kg not only facilitated the polarization of microglia toward the M2 phenotype but also effectively suppressed the release of proinflammatory cytokines.

However, limited research has been conducted on the synergistic effects of these 2 treatments in regulating neuroinflammation during the acute phase following ischemic stroke. In this study, we provided evidence that the combination of curcumin delivery nanosystem and TLUS exerts an outstanding regulatory influence on microglia polarization balance and neuroprotection, surpassing the efficacy of these 2 treatments administered independently. The synergistic effect may be partly attributed to ultrasound facilitating the transport of curcumin across the BBB, as well as enhancing material exchange across the cell membrane, thereby promoting cellular uptake of curcumin. Further research is required to confirm these hypotheses [54]. The integration of TLUS with other nanocarriers also presents potential advantages. Exosomes possess high bioavailability, stability, low toxicity, and low immunostimulation and can also be modified to facilitate targeted drug delivery [55]. However, the targeted delivery of exosomes to the brain remains a challenging endeavor due to the presence of the BBB [56]. Recent studies have demonstrated that TLUS can transiently, reversibly, and locally disrupt the BBB, thereby facilitating the targeted delivery of exosomes to specific regions of the brain. However, current research has predominantly focused on applications in glioma [57] and Alzheimer’s disease [58], with relatively limited investigation into its potential use in cerebral ischemia. In conclusion, further research is required to validate the therapeutic efficacy of TLUS combined with other drug delivery systems in the context of cerebral ischemia [59].

Through RNA sequencing and in vitro experiments, we ultimately demonstrated that synergistic treatment predominantly promotes microglial M2 polarization by inhibiting the MAPK and NF-κB signaling pathways. As a pivotal signaling pathway orchestrating neuroinflammation, the widespread activation of MAPK after cerebral stroke not only triggers an excessive production of proinflammatory mediators but also exerts a profound influence on cell survival and proliferation [60]. The suppression of the NF-κB signaling pathway, leading to a subsequent reduction in proinflammatory mediators and ROS production, is also regarded as a highly promising therapeutic approach for mitigating the detrimental effects of cerebral ischemia [61]. However, there are some limitations in this research. First, the influence of varying formulation ratios of RRP@Lipo-Cur on its precise functional outcomes has not yet been determined. Therefore, the exploration investigating optimal ratio of different components to maximize its biological functional effect is still required. Second, only young male mice were included in study, and the potential influence of sex and age on treatment efficacy was not investigated. Third, the impact of synergistic therapy on the long-term outcomes following ischemic stroke warrants further investigation.

In summary, this study introduces RRP@Lipo-Cur as a novel and promising nanodrug delivery system, enhanced by the controlled release mechanism and synergistic therapeutic effects of TLUS. This approach integrates the advantages of nanobiological materials and physiotherapy agents, offering a promising solution for the treatment of ischemic stroke. RRP@Lipo-Cur and TLUS combination therapy exhibits exceptional biocompatibility, precise targeting to lesioned brain regions, and a significant neuroinflammation modulatory effect, overcoming the inherent limitations of conventional pharmacotherapy. This study aims to introduce a novel concept that integrates nanodrug delivery system with physical agent therapy. This approach not only emphasizes the intrinsic therapeutic efficacy of the drug but also leverages the characteristics of ultrasound to enhance the functionality of nanomaterials and the therapeutic impact of the drug, which provide therapeutic strategies for the targeted and noninvasive treatment of other brain diseases.

## Conclusion

A neuron-targeted and ROS- and ultrasound-responsive smart drug delivery liposome loaded with curcumin was developed to address cerebral ischemia–reperfusion injury. RRP@Lipo-Cur could efficiently target brain and facilitate liposome retention in the ischemic brain areas, while high ROS levels and ultrasound stimulation promote drug release. Notably, the synergistic treatment of RRP@Lipo-Cur and TLUS significantly facilitated the polarization of M1 to M2 microglial cells following CIRI via the MAPK and NF-κB pathways. This process effectively promoted the establishment of an anti-inflammatory microenvironment in the ischemic penumbra, thereby providing neuroprotection for the damaged neurons. At the same time, the experimental data demonstrated that the efficacy of synergistic treatment was significantly superior to that of individual treatment. In summary, the synergistic treatment of RRP@Lipo-Cur and TLUS demonstrated remarkable therapeutic effects in regulating the inflammatory microenvironment within the ischemic penumbra, enhancing neuronal survival, and improving neurological function. The synergistic treatment approach combining nanobiological materials with therapeutic ultrasound offers an innovative alternative for the management of neurological disorders.

## Methods

### Preparation of RRP@Lipo-Cur

First, 1,2-dioleoyl-sn-glycero-3-phosphoethanolamine (DOPE), cholesterol, DSPE-TK-mPEG-RVG29 (Xian Ruixi, China) were dissolved in 5 ml of chloroform, and PpIX (Xian Ruixi, China) and curcumin were dissolved in a small amount of methanol and evaporated into a film in a sample bottle under reduced pressure, adding 10 ml of deionized water. After adding deionized water, it was treated with ultrasound and liposome extruder (polycarbonate membrane, pore size 100 nm) (Fig. 2A). Nanodialysis apparatus was used for dialysis (polycarbonate membrane, pore size 50 nm). Then, deionized water was added to 20 ml. Finally, freeze-protectant was added and freeze-dried. When used, an appropriate amount of solid dry powder was taken as needed, and pure water was added until the lyophilized powder was completely dissolved. The water bath ultrasound can be used to promote the dispersion of nanomaterials by 5 to 30 s. Dynamic light scattering (Nano-ZS90, Malvern, UK) was used to measure the liposomes’ particle size and ζ-potential, while TEM (FEI, USA) was used to assess their morphologies. High-performance liquid chromatography was used to measure LC and EE (Shimadzu, Japan). UV-Vis absorption spectrometer (Beijing Puxi, China) and infrared spectrometer (Thermo Scientific, USA) were used to measure absorbance and penetration rate of RRP@Lipo-Cur, respectively.

### In vitro release

In the in vitro drug release assay, we quantified the concentrations of PpIX and curcumin using UV spectrophotometry at specific time points. The procedure is as follows: At predetermined time intervals, aliquots of the liposome dispersion were withdrawn from the dialysis device. These samples were subsequently extracted with Triton to disrupt the liposomal structure and facilitate the release of the encapsulated drugs. The aqueous phase was then isolated, and absorbance measurements were conducted at the respective maximum absorption wavelengths for each compound using a UV spectrophotometer. Specifically, curcumin exhibited a maximum absorption wavelength of approximately 470 nm, while PpIX showed a peak at around 350 nm. Drug concentrations in the samples were determined by referencing standard calibration curves. By measuring drug concentrations at various time points, cumulative release profiles were generated.

### Treatment and groups in vitro

RRP@Lipo-Cur intervention started at the end of OGD (beginning of reoxygenation) for 6 h to simulate the intervention model after CIRI. After 6 h of treatment, the culture medium was discarded, and cells were washed 3 times with PBS and reoxygenated for more 18 h with new culture medium. RRP@Lipo-Cur (200 μg/ml) was decided as the optimal concentration for cell experiment through the cell counting kit-8​ (CCK8) assay (Fig. S1). Cells in groups that required TLUS treatment were conducted as follows. The transcranial ultrasound parameters were used at a frequency of 1 MHz, a pulse of 100 Hz, 50% duty cycle, and an intensity of 300 mW/cm^2^ SATP (spatial average temporal peak). The specific operation method is to apply the coupler to the bottom of the culture dish with the fixation method, as described in our previous study [62]. Ultrasound was administered twice during the RRP@Lipo-Cur intervention, for 5 min at the beginning of the addition of RRP@Lipo-Cur, and for another 5-min treatment after 3-h interval. In summary, the control group received addition of saline and stimulation of TLUS in the off state; the OGD/r group received OGD/r operation, addition of saline, and stimulation of TLUS in the off state; the TLUS group received OGD/r operation, addition of saline, and 300 mW/cm^2^ TLUS stimulation; the RRP@Lipo-Cur group received OGD/r operation, addition of RRP@Lipo-Cur, and stimulation of TLUS in the off state; the RRP@Lipo-Cur + TLUS group received OGD/r operation, addition of RRP@Lipo-Cur, and 300 mW/cm^2^ TLUS stimulation.

### Treatment and groups in vivo

 Detailed methodological steps for applying TLUS in mice are outlined below. The transcranial ultrasound parameters were determined at a frequency of 1 MHz, a pulse of 100 Hz, 50% duty cycle, and an intensity of 300 mW/cm^2^ SATP, and the diameter of the collimator is 5 mm with an effective radiating area of 4 mm^2^. The operation method of TLUS stimulation was shown in Fig. S5. According to the biodistribution result, we found that RRP@Lipo-Cur accumulation in mouse brain was highest 6 h after tail vein injection (Fig. 4E). Therefore, the timing of ultrasound treatment was determined to be 6 h after injection for a total of 3 times. Mice that required TLUS treatment received 2 stationary ultrasound stimuli to entirely cover the infarct area, each lasting 5 min. As for RRP@Lipo-Cur administration, mice that required RRP@Lipo-Cur received 300 mg/kg injection of RRP@Lipo-Cur 24 h after MCAO/r surgery. The dosage was determined through dose-escalation behavioral trials in Fig. S2. RRP@Lipo-Cur were given every 48 h for a total of 3 times until sacrifice. In summary, the Sham group received sham operation, tail intravenous injection of saline, and stimulation of TLUS in the off state; the MCAO/r group received MCAO/r operation, tail intravenous injection of saline, and stimulation of TLUS in the off state; the TLUS group received MCAO/r operation, tail intravenous injection of saline, and 300 mW/cm^2^ TLUS stimulation; the RRP@Lipo-Cur group received MCAO/r operation, tail intravenous injection of RRP@Lipo-Cur, and stimulation of TLUS in the off state; the RRP@Lipo-Cur + TLUS group received MCAO/r operation, tail intravenous injection of RRP@Lipo-Cur, and 300 mW/cm^2^ TLUS stimulation.

### Animal

Shanghai Jihui Laboratory Animal Care Co. Ltd. supplied the adult male C57BL/6J mice (20 to 25 g), which were kept in a 12-h/12-h light/dark cycle (lights on at 7:00 AM), at a constant room temperature (RT) (22 ± 2 °C), with food and water available at all times for at least 1 week prior to the experiments. Every experiment was performed in compliance with the standards established by the Huashan Hospital’s Animal Care and Use Committee at Fudan University (approval number: 202407016S). Every attempt was made to prevent or lessen animal suffering. At same time, the number of animals utilized in the project was minimized through rational experimental design and a sterile surgical environment.

### Cell culture

The Shanghai Institute of Biological Sciences, Chinese Academy of Sciences, provided the mouse hippocampal neuron cell line (HT-22), the brain-derived endothelial cell line (bEND.3), and mouse microglia cells (BV-2). The cells were maintained at 37 °C in a humidified incubator with 5% CO_2_ in Dulbecco’s modified Eagle’s medium (DMEM) (Gibco, USA), supplemented with 10% heat-inactivated fetal bovine serum (FBS) (Gibco, USA), 100 μg ml^−1^ streptomycin, and 100 U ml^−1^ penicillin (Yesen, China).

### MCAO/r

The MCAO/r mouse model was developed as follows. The mouse was positioned in a supine position and kept immobile following anesthesia, hooking the incisors with sutures and gently stretching the neck to fully expose the surgical area. A heating pad was used to stabilize the mice’s body temperature at 37 °C so as to maintain their physiological body temperature. Subsequently, the skin was incised along the midline in the neck of the mouse, and the left common carotid artery, external carotid artery, and internal carotid artery were bluntly separated. MCAO/r monofilament (diameter: 0.18 ± 0.01 mm, Guangzhou Jialing Co. Ltd., China), with its head end coated with soft silicone, was inserted through the stump of the internal carotid artery and advanced to a depth of about 10 mm until slight resistance was encountered, thereby occluding the left middle cerebral artery. After the occlusion was maintained for 1 h, the monofilament was carefully pulled out to reestablish the blood supply and produce reperfusion injury. Only skin and subcutaneous tissue incisions were made in the Sham group, and no further operation was done.

### OGD/r treatment

BV-2 and HT-22 cells were treated with OGD/r to model CIRI in vitro as follows. Cells were first rinsed 3 times with PBS (Servicebio, Wuhan) and then incubated in glucose-free DMEM (Gibco) without glucose and FBS at 37 °C. Next, the cells were moved to an anaerobic incubator containing 95% N_2_ and 5% CO_2_ (37 °C) to create OGD conditions. After 4 h of OGD treatment, the cells were cocultured with conventional DMEM containing glucose and 10% FBS and moved to a normoxic incubator for 24 h to construct the OGD/r model.

### TTC staining

The TTC staining was used to quantify the ischemic infarction of MCAO/r mice with different treatments. Briefly, after euthanization, the brains of mice were immediately collected and then coronally cut into six 2-mm-thick slices with a brain mold. Then, slices were stained with 2% TTC solution for 30 min at 37 °C (Solarbio, China) and then fixed in 4% paraformaldehyde (PFA). Images were analyzed using ImageJ software [National Institutes of Health (NIH), USA] to obtain ischemic infarct area. The infarcted volume was normalized and displayed as a percentage of the non-ischemic hemisphere to compensate for edema. That is the ratio of (Contralateral non-infarct size minus Ipsilateral non-infarct size) to (Contralateral non-infarct size * 2). The details of the quantification method were refereed to our previous research [35].

### Nissl staining

Nissl staining was carried out as previously described. In detail, the tissue sections were deparaffinized by immersion in graded ethanol solutions (70%, 95%, and 100%) for 3 min each, followed by rehydration through descending concentrations of ethanol (95%, 70%, and 50%) for 3 min each. Afterward, the sections were stained with 0.1% toluidine blue solution for 20 min, briefly rinsed with distilled water, and subsequently differentiated in 95% ethanol for 15 min. Last, 3 fields were randomly selected from the ischemic peripheral zone of each mice brain, and the number of Nissl bodies in each field was recorded and subsequently averaged. Nissl bodies among 5 groups were statistically analyzed.

### Enzyme-linked immunosorbent assay

A microplate reader (Thermal, USA) was used to measure the absorbance of ELISA plates at 450 nm after the background was eliminated at 650 nm. Following the manufacturer’s instructions, ELISA kits (Laizi, China) were used to measure the concentrations of IL-1β, TNF-α, IL-4, and IL-10 from 3 ischemic penumbra samples and 3 supernatants of BV-2 in each group.

### Protein extraction and preparation

Brain tissue from the ischemic penumbra of parietal cortex and the comparable region on the same side in healthy mice was prepared. Radioimmunoprecipitation assay lysis buffer (Beyotime, China) paired with a protease inhibitor and a phosphatase inhibitor (Beyotime, China) was utilized to obtain cell lysates from BV-2 cells, HT-22 cells, and aimed brain tissue of mice. For specific condition, nuclear and cytoplasmic extraction reagents (Beyotime, China) were used for collecting nuclear and cytoplasmic extracts following the manufacturer’s instructions. Before WB analysis, concentrations of protein were measured with an improved bicinchoninic acid (BCA) protein assay kit (Beyotime, China).

### WB analysis

When protein samples were ready, 30 μg of proteins from each sample was electrophoresed in a 10% sodium dodecyl sulfate–polyacrylamide gel electrophoresis (SDS-PAGE) gel (Epizyme Biotech, China) at voltages of 90 V for 15 min and 120 V for 60 min. Then, the separated protein was transferred onto a polyvinylidene difluoride (PVDF) membrane (Millipore, USA) at constant current of 400 mA for 60 min. The membranes were treated overnight at 4 °C with the appropriate primary antibody diluted in blocking solution after being blocked with 5% bovine serum albumin (BSA) for 1 h at RT. Primary antibodies were listed in Table S1. Secondary antibodies, which were horseradish peroxidase (HRP)-conjugated goat anti-mouse or anti-rabbit immunoglobulin G (IgG) at concentration of 1:10,000 (Yesen, China), were incubated for 1 h at RT after 3 washes with TBS-T buffer (10 mM tris, 150 mM NaCl, 0.05% Tween 20, pH 7.5). After another 3 washes, the blots were exposed with enhanced chemiluminescence (ECL) reagents (Biosharp, China) and imaged with the ChemiDoc Touch Chemiluminescence Imaging System (Bio-Rad, USA). After being normalized with the loading control (β-actin), the relative gray scale value was quantitatively evaluated using ImageJ software (NIH, USA).

### Modified neurological severity score

At 24 h and 7 d after MCAO/r surgery, the neurological functional outcome was assessed using the mNSS test. The whole mNSS score comprises 14 points, which includes tests for reflexes, balance, and motor function (muscle condition and abnormal movement). Severe, moderate, and mild injuries are indicated by cumulative ratings of 10 to 14, 5 to 9, and 1 to 4, respectively. One point will be given for failing to complete one of the tests, and one point will be deducted for no corresponding test reflection. Blind independent examiners conducted the mNSS test.

### Open-field test

Mice were placed in an open-field cubic box (40 × 40 × 40 cm^3^), and their locomotor activity was recorded using the motion trajectory monitoring system. For 10 min after habituation, the animals’ overall locomotor activity was monitored. Between the 2 tested mice, the box was completely cleaned with 75% ethanol. We recorded and analyzed time of immobility, average speed, crossing times into the central area, total distance explored, and distance in the central area to collect general activity characteristics of mice in each group.

### Rotarod test

Rotarod test was performed 24 h and 7 d after MCAO/r surgery. Within 90 s, the rod’s speed increases from 5 rpm to 40 rpm. Then, it maintains a consistent rotational speed. To prevent exhaustion, there is a 10-min break between each 5-min exam. A fall from the rotarod was ​also defined as​ the mouse passively completing 3 full rotations along with the rod. This was repeated 3 times for each mouse, and the average of 3 latency times was taken.

### CatWalk XT gait test

To collect gait data, a high-speed camera was positioned beneath the glass track of the CatWalk XT system (Noldus, Netherlands) to obtain images of the mouse’s paw. In the experiment, mice were placed on a racetrack to pass smoothly and were required to do so within 2 to 6 s. At least 3 gaits were recorded for each mouse tested. The gait analysis indicators presented in the study included swing time, stand time, footprint mean intensity, average speed, and 2D gait cycle.

### Transmission electron microscopy

After anesthesia was administered to mice in different treatment groups, precooled PBS perfusion was rapidly performed, and then the ischemic penumbra of left cerebral hemisphere of the mice were immediately removed and fixed in 2.5% glutaraldehyde. The ischemic penumbra tissue was removed and fixed overnight (4 °C) in 2.5% glutaraldehyde. The fixed tissue sample was transferred to 1% OsO_4_ for 2 h. Next, the samples were washed 3 times with cacodylate buffer. After washing, the samples were stained in 1% uranyl acetate for 1 h. At the end of staining, the samples were sequentially dehydrated in fractionated ethanol solutions, and finally, the dehydrated samples were embedded in Epon. The sections were cut on an ultramicrotome (Leica) and placed on an transmission electron grid. To enhance contrast, sections were double-poststained with uranium acetate and lead citrate. The sections were observed and examined under a Hitachi 7100 transmission electron microscope (Nikon). The condition of the mitochondria located in proximity to the nucleus was examined.

### Experiments with BV-2 conditioned medium

As shown in Fig. 8A, 4 h after OGD treatment, the culture supernatant from BV-2 cells was replaced with complete medium and different treatments were performed at the same time. In the control group, cells without OGD treatment were treated with PBS only, while the groups with OGD received the following 4 interventions at the start of reperfusion: PBS addition, TLUS treatment, RRP@Lipo-Cur addition, and TLUS + RRP@Lipo-Cur treatments. TLUS treatment received twice 5-min ultrasound exposure in every 3 h. Then, after 6 h of treatment, the culture medium was replaced and the reoxygenation treatment was continued for 18 h, for a total of 24 h of reoxygenation. After reoxygenation, the supernatant of BV-2 in different groups was collected as BV-2 conditioned medium (BCM) and cocultured with HT-22 cells for 24 h. The first group is normal HT-22 cocultured with BCM from non-OGD- and PBS-treated BV-2 cells. The second group is OGD/r HT-22 cocultured with BCM from non-OGD- and PBS-treated BV-2 cells. The third group is OGD/r HT-22 cocultured with BCM from OGD- and PBS-treated BV-2 cells. The fourth group is OGD/r HT-22 cocultured with BCM from OGD- and ultrasound-treated BV-2 cells. The fifth group is OGD/r HT-22 cocultured with BCM from OGD- and RRP@Lipo-Cur-treated BV-2 cells. The sixth group is OGD/r HT-22 cocultured with BCM from OGD- and TLUS + RRP@Lipo-Cur-treated BV-2 cells. After 24 h of reoxygenation, the HT-22 cells were observed and analyzed.

### Apoptosis assessment in vitro

To access the therapeutic effect of different treatments on cell apoptosis rate after OGD/r, the Annexin V–FITC (fluorescein isothiocyanate)/PI (propidium iodide) apoptosis detection kit (Beyotime, China) was performed. Then, after 24 h of reperfusion, cells were washed 3 times with PBS and collected via centrifugation. Next, the cells were stained with 10 μl of Annexin V-FITC and 5 μl of PI for 15 min. Finally, the cells were analyzed using flow cytometry to accurately assess the degree of apoptosis. In addition, Live/Dead staining was also performed according to the manufacturer’s instructions (Beyotime, China). First, HT-22 cells were cleaned with PBS twice after cell culture medium was removed. Then, the cells were cultured with working solution for 30 min at a temperature of 37 °C. Last, the working solution was replaced with PBS, and cells were observed under a fluorescence microscope (excitation/emission = 494/517 nm for calcein AM and 535/617 nm for PI). Apoptosis rate was obtained by calculating the ratio of PI-stained cells to the total number of cells.

### Immunofluorescence staining

Mice were perfused with saline through the heart after anesthesia, followed by 4% PFA for fixation. Brain tissue was quickly removed and fixed overnight with 4% PFA at 4 °C. The tissues were sequentially immersed in different concentrations of ethanol solution and gradually dehydrated. This was followed by immersion in xylene to remove residual ethanol and make the tissue transparent. Finally, it was embedded with paraffin. A slicer was used to cut the implanted brain tissue into slices that were 5 μm thick. The sections were then hydrated using a series of graded ethanol and passed through xylene to remove the paraffin. Sections were then subjected to antigen retrieval in citrate buffer.​ After washing in PBS, sections were permeabilized with 0.5% Triton X-100 (Solarbio, China) and incubated with 5% normal goat serum for 1 h at RT. For cells, 4% PFA was used to fixed cells for 15 min at RT after discarding the culture medium and 3-time PBS wash. Then, cells were incubated with 0.15% Triton X-100 for 15 min to permeabilize the cell membrane and 3% BSA for 1 h. They were then incubated with primary antibody (listed in Table S1) overnight at 4 °C. The next day, after recycling primary antibody and 3-time phosphate buffered saline with Tween 20 (PBST) wash, the sections or cells were incubated with fluorescent secondary antibody for 1 h at RT. Finally, the operation of 4′,6-diamidino-2-phenylindole (DAPI) staining and sealing the slides was performed. Immunoreactivity was photographed through a fluorescence microscope (ECHO Revolve, USA).

### Flow cytometry in vivo

Flow cytometry is an effective technique for assessing the expression of multiple markers and distinguishing between different cell populations in the brain. Mice were perfused with saline through the heart after anesthesia to eliminate the influence of circulating macrophages. The mouse brain was cut with small scissors to allow for cell dispersion. Next, the prepared cell suspension was passed through a 70-μm pore size nylon mesh (Millipore, USA) in a 50-ml tube and gently pushed with a syringe plunger. After that, the suspension was dispersed in PBS and carefully placed on 30% and 70% Percoll gradients (Solarbio, China). The medulla and erythrocytes were removed by centrifugation at 1,500*g* for 20 min at RT. The cell layer located between 30% and 70% Percoll gradients was collected and blocked with 1% FBS to reduce nonspecific binding, followed by staining with fluorescently labeled antibodies. To specifically analyze macrophages and microglia, cells were stained with the following antibodies (listed in Table S1). Finally, the cells were analyzed using flow cytometry (BD Biosciences, USA) to accurately assess cell populations.

### TUNEL staining

To explore the neuroprotective effect on neurons, TUNEL staining was used in this research. At day 7, mice were anesthetized and sacrificed. The brain tissue sections were fixed with 4% PFA, permeabilized with 0.1% Triton X-100 solution, and then blocked with 3% BSA for 1 h. Brain sections were stained with One-Step TUNEL Apoptosis Assay Kit (Servicebio, China), according to the manufacturer’s guidelines. The sections were observed and analyzed using a spinning disk confocal super-resolution microscope (ECHO Revolve, USA). The percentage of dead cells was counted by calculating the ratio of TUNEL and DAPI double-stained cells to the total number of DAPI-stained cells.

### Safety evaluation in vivo

After multiple administration experiments, we evaluated the safety of RRP@Lipo-Cur by measuring serum concentrations of CREA, ALT, AST, UA, and BUN. In addition, H&E staining technology was used to visualize the details of cell and tissue structure to more clearly observe and analyze their pathological changes.

### Flow cytometry in vitro

To induce M1 microglia polarization, BV-2 was treated with OGD for 4 h and reoxygenation for 24 h. The treatment was introduced at the start of reoxygenation for 6 h and then replaced with new culture medium for 18 h. Then, HT-22 was collected for flow cytometry analysis after staining with flow cytometric antibody. BV-2 was treated for 24 h with IL-4 (40 ng/ml) to induce M2 microglia polarization. The treatment was introduced after 24-h IL-4 intervention for 6 h. Then, HT-22 was collected for flow cytometry analysis after staining with flow cytometric antibody (listed in Table S1).

### CSF1R inhibitor treatment

Microglia depletion was performed as previously described. Mice were given the CSF1R inhibitor PLX3397 (AdooQ BioScience, A15520) at a dose of 290 mg/kg in AIN-76A standard chow. The control group received AIN-76A standard chow only. Before MCAO/r was induced, mice were given the chow diet freely for 3 weeks, and they continued feeding on it until they were euthanized.

### Cellular uptake study

bEND.3 and HUVECs were seeded in 12-well plates at a density of 2 × 10^5^ cells per well for 24 h. The cell culture media were changed with 1 ml of fresh media containing DiD-RRP@Lipo-Cur. After 30-, 60-, 90-, and 120-min incubation, cells were stained with DAPI and then photographed by a laser scanning confocal microscope. Cells incubated for 30 and 120 min with DiD-RRP@Lipo-Cur were also collected for further analysis of DiD intensity via a flow cytometer (BD FACSCelesta, USA).

### Biodistribution in MCAO/r mice

Tail veins of MCAO/r mice were injected with DiD or DiD-RRP@Lipo-Cur. The Caliper IVIS Lumina III In Vivo Imaging System (Perkin Elmer, USA) was used to detect fluorescence intensity in vivo at certain times (1, 2, 6, 12, and 24 h) to assess the biodistribution of DiD in the brain. One batch of mice was sacrificed 6 h post-injection, and their brains were harvested for imaging analysis. Brains were also performed with immunofluorescent staining to detect DiD intensity in the brain. The fluorescence intensity was measured using ImageJ (NIH, USA).

### Statistical analysis

The data are presented as mean ± SD unless otherwise specified. For comparisons between 2 groups, a 2-tailed unpaired Student’s *t* test was employed. To compare multiple groups, one-way analysis of variance (ANOVA) was utilized, followed by the Bonferroni post-hoc test to assess significant differences between any 2 groups. Statistical significance was defined as *P* < 0.05. All statistical analyses were performed using GraphPad Prism version 9.0.

## Data Availability

All data of this study are available from the corresponding authors upon reasonable request.
