# Supplementary material for: Synergistic Therapeutic Platform Combining Transcranial Low-Intensity Ultrasound Stimulation and Curcumin Load Liposome Ameliorates Cerebral Ischemia by Modulating Microglia Polarization-Mediated Neuroinflammatory Microenvironment
Source: Research (Wash D C). 2025 Sep 11;8:0861. doi: 10.34133/research.0861 (PMC12423505; doi:10.34133/research.0861)
Supplement: Supplementary 1 — Figs. S1 to S16 Tables S1 to S4 [file research.0861.f1.zip › Supplemental Figure.docx]

**Synergistic Therapeutic Platform Combining TLUS and Multi-Functional Curcumin-Load Liposome Ameliorate Cerebral Ischemia by Modulating Microglia Polarization-Mediated Neuroinflammatory Microenvironment**

**
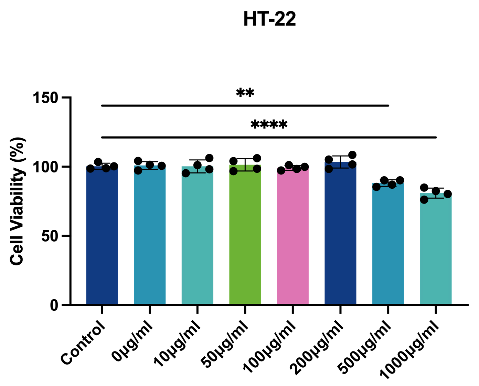
**

**Figure S1. Cell viability of HT-22 after treatment with** **RRP@Lipo-Cur at different concentrations. (n=4) **p<0.01** ******p<0.0001.**


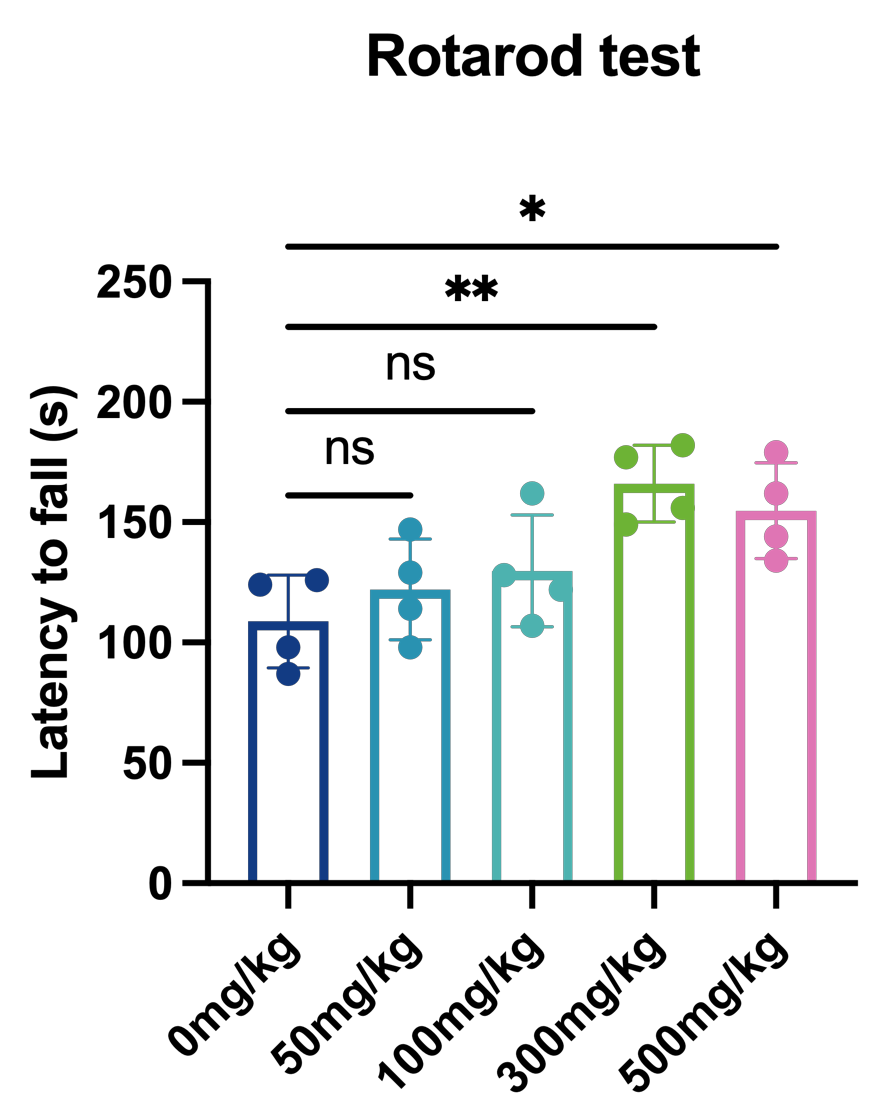


**Figure S2.** **Dose-escalation trials of RRP@Lipo-Cur in MCAO mice via tail vein injection. (n=4). ns>0.05, *p<0.05, **p<0.01.**

**
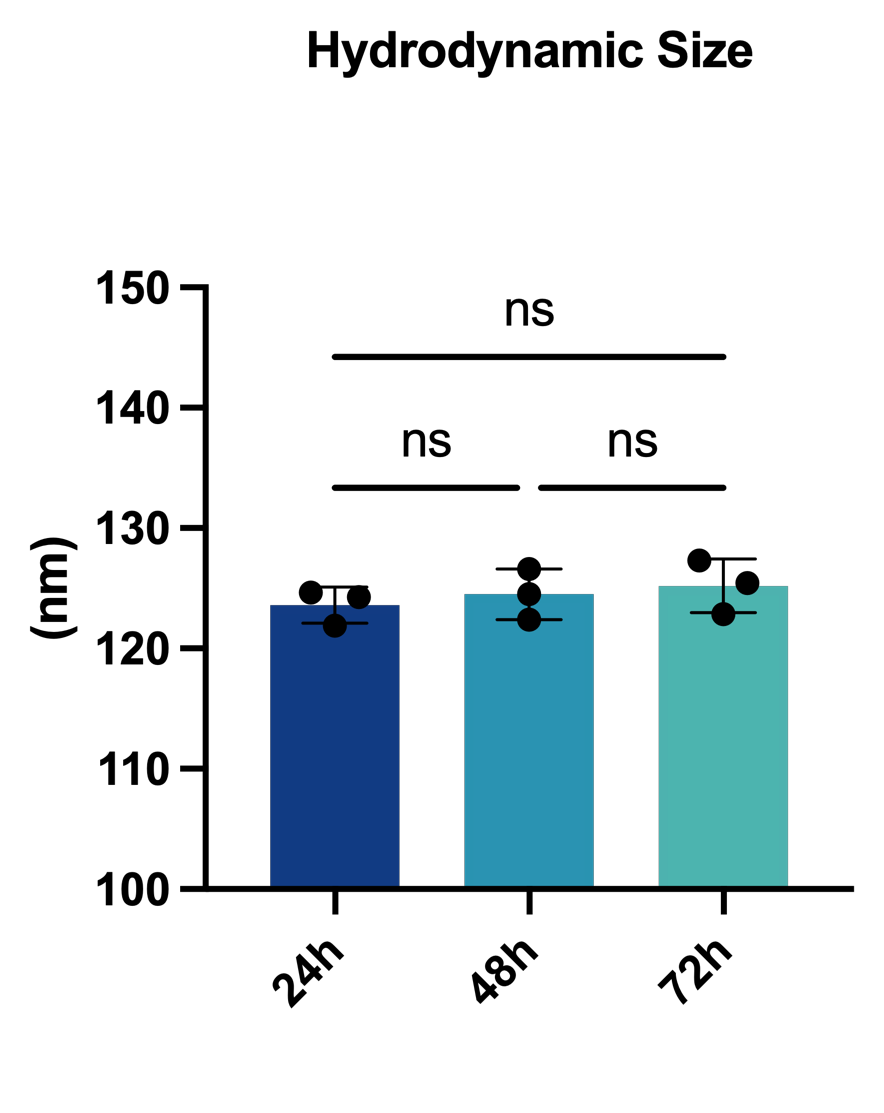
**

**Figure S3. Hydrodynamic size of RRP@Lipo-Cur after dissolving in PBS after 24h, 48h, and 72h. (n=3) ns>0.05.**

**Figure S4.** **Statistical plot of cumulative release of curcumin and PpIX in H_2_O_2_ and PBS at different time points. (n=3) ns>0.05; ********p<0.0001; ***p<0.001;**


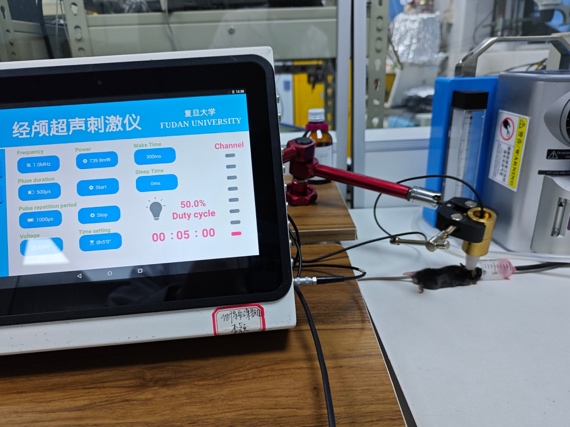

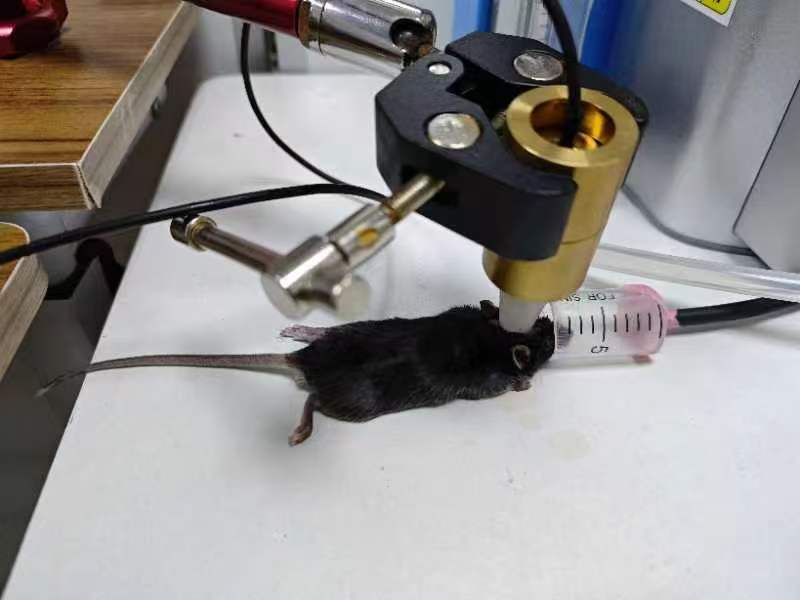


**Figure S5. Operation procedure of TLUS in mice**

**Figure S6. Apoptosis rate of HT-22 cells after 300mW/cm^2^ transducer output intensity**

**Figure S7. Analysis for therapeutic effect of unloaded nanomaterials (RRP@Lipo) on the apoptosis of neurons in ischemic penumbra and infarction after ischemia-reperfusion in mice. (A-B) TTC staining comparing effects of different groups (Control group, MCAO/r group, and RRP@Lipo group) on cerebral infarction (Infarcted regions: white; Normal tissue: red). The infarct areas across groups were calculated. (n=3) ns>0.05 ***p<0.001.** **(C-F) The protein expression of Bcl-2, Cleaved-Caspase 3, and Bax in ischemic penumbra tissue across three groups were analyzed by Western Blot. (n=3) ns>0.05 ***p<0.001 ****p<0.0001.**

**Figure S8. Analysis for therapeutic effect of Free Curcumin+TLUS (RRP@Lipo) on the apoptosis of neurons in ischemic penumbra and infarction after ischemia-reperfusion in mice to prove the necessity of the nanocarrier. (A-B) TTC staining comparing effects of different groups (MCAO/r group, RRP@Lipo^Cur^+TLUS group, and Cur+TLUS group) on cerebral infarction (Infarcted regions: white; Normal tissue: red). The infarct areas across groups were calculated. (n=3) *p<0.05 ***p<0.001. (C-F) The protein expression of Bcl-2, Cleaved-Caspase 3, and Bax in ischemic penumbra tissue across three groups were analyzed by Western Blot. (n=3) *p<0.05 **p<0.01 ***p<0.001.**


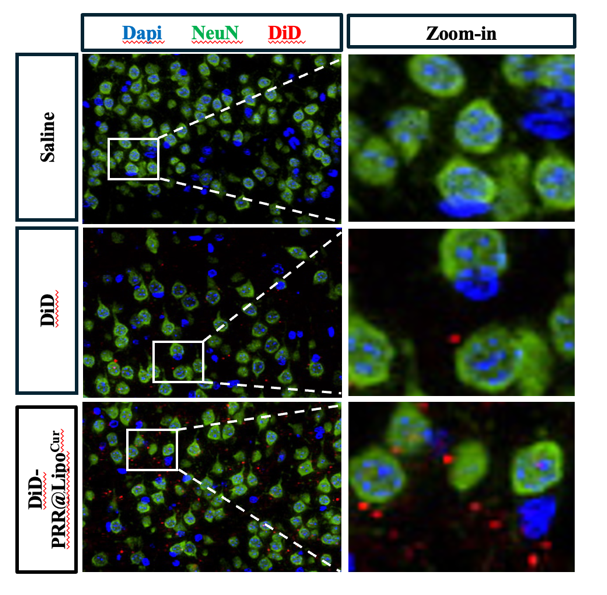


**Figure S****9. Fluorescence imaging showed co-localization of DiD with NeuN (Green: Neuron marker) in the ischemic penumbra area of MCAO/r mice to demonstrate the affinity of RRP@Lipo-Cur to CNS**.

**Figure S10. Additional charts of RNA-seq**

**(A)** **RNA-seq showed 5673differentially expressed genes with 3228 genes up-regulated, 2445 genes down-regulated. (B-C) Heatmap of gene expressions associated with "regulation of immune system process", "programmed cell death" across two groups. (D-E) GEAS analysis and significantly changed DEGs for "negative regulation of neuron apoptotic process" and "neuroinflammatory response" after combinative treatment.**

**Figure S11. RRP@Lipo-Cur+TLUS was unable to significantly ameliorate OGD/r-induced cell apoptosis of HT-22**

**(A-B) Flow cytometry analysis for apoptosis rate in HT-22 cells across five groups. (n=3) ns >0.05 ****p<0.0001. (C-D)** **Apoptosis related protein expression among five groups. (n=3)** **ns>0.05 ***p<0.001 ****p<0.0001.**

**Figure S12: Combined therapy decreased** **pro-inflammatory Cytokines expression and increased anti-inflammatory OGD/r-induce BV-2**

**(A) ELISA analysis was used to detect concentration of** **extracellular factors.** **(n=4) ns>0.05 *p<0.05 **p<0.01 ***p<0.001 ****p<0.0001. (B-C) Intracellular factors expression among five groups. (n=3) ns>0.05 ***p<0.001 ****p<0.0001.**

**Figure S13. Expression of inflammatory cytokines in the ischemic penumbra of MCAO/r mice receiving different treatments. (n=4) ns>0.05 *p<0.05 **p<0.01 ***p<0.001 ****p<0.0001.**

**Figure S14: Behavioral test after PLX3397 chow**

**(A-B)** **Representative trajectory illustration of OPT. Move distance and crossing time were recorded and statistically analyzed between Control chow group and PLX3397 group. (n=3) ns>0.05. (C) Representative gait 2D pattern of two group.**

**Figure S15. TNF-α related Heatmap and GSEA analysis for neuron apoptosis and neuroinflammation.**

**(A-B) Heatmap of DEGs associated with “Cytokine-cytokine receptor interaction”and “TNF signaling pathway” across two groups. (C-D) GSEA analysis was enriched in “tumor necrosis factor superfamily cytokine production” and “tumor necrosis factor mediated signaling pathway”.**

**Figure S16. Safety of** **RRP@Lipo-Cur**

**(A)Analysis of ALT and AST levels in mouse blood following various treatments. (n=3) ns>0.05. (B) Analysis of BUN, CREA, and UA levels in mouse blood following various treatments. (n=3) ns>0.05. (C) HE staining for heart, kidney, spleen, lung, and liver after 3-time RRP@Lipo-Cur or saline treatment.**
