# Supplementary material for: Synergistic Therapeutic Platform Combining Transcranial Low-Intensity Ultrasound Stimulation and Curcumin Load Liposome Ameliorates Cerebral Ischemia by Modulating Microglia Polarization-Mediated Neuroinflammatory Microenvironment
Source: Research (Wash D C). 2025 Sep 11;8:0861. doi: 10.34133/research.0861 (PMC12423505; doi:10.34133/research.0861)
Supplement: Supplementary 1 — Figs. S1 to S16 Tables S1 to S4 [file research.0861.f1.zip › Supplemental Table 1 and 2 .docx]

**Supplemental Table 1. Cumulative release of Curcumin and PpIX in PSB and 5mM H_2_O_2_**

| **PpIX** | PBS |  |  |  |
| --- | --- | --- | --- | --- |
|  | Time（h） | Release rate% |  |  |
|  | 0 | 0 | 0 | 0 |
|  | 1 | 28.9 | 30.1 | 29 |
|  | 2 | 38.5 | 37.6 | 38.4 |
|  | 4 | 47.2 | 47.4 | 47.5 |
|  | 6 | 50.3 | 50.9 | 51.8 |
|  | 24 | 78.2 | 78.6 | 79.9 |
|  | 48 | 88.9 | 89.2 | 89.5 |
|  |  |  |  |  |
|  | H_2_O_2_ |  |  |  |
|  | Time（h） | Release rate% |  |  |
|  | 0 | 0 | 0 | 0 |
|  | 1 | 47.6 | 46.6 | 47.8 |
|  | 2 | 56.8 | 57.2 | 57.3 |
|  | 4 | 73.4 | 73.5 | 74.2 |
|  | 6 | 79.9 | 80.1 | 80 |
|  | 24 | 89.5 | 89.7 | 90.5 |
|  | 48 | 94.3 | 94.2 | 94.8 |
|  |  |  |  |  |
| **Curcumin** | PBS |  |  |  |
|  | Time（h） | Release rate% |  |  |
|  | 0 | 0 | 0 | 0 |
|  | 1 | 30.8 | 31.7 | 30.9 |
|  | 2 | 39.7 | 41.3 | 41.8 |
|  | 4 | 47.9 | 47.1 | 46.6 |
|  | 6 | 53.3 | 53.4 | 54.5 |
|  | 24 | 80.5 | 81.5 | 80.9 |
|  | 48 | 89.4 | 89.5 | 91.3 |
|  |  |  |  |  |
|  | H_2_O_2_ |  |  |  |
|  | Time（h） | Release rate% |  |  |
|  | 0 | 0 | 0 | 0 |
|  | 1 | 42.3 | 43.5 | 42.4 |
|  | 2 | 51.9 | 52.2 | 52.3 |
|  | 4 | 67.7 | 67.5 | 67.9 |
|  | 6 | 74.5 | 74.9 | 76.2 |
|  | 24 | 85.7 | 85.3 | 86.9 |
|  | 48 | 90.7 | 92.3 | 91.8 |

**Supplemental Table 2. Primary antibodies used in the experiment**

| Antibody | Source | Catalog No. | Type | Dilution | M.W. (kD) |
| --- | --- | --- | --- | --- | --- |
| iNOS | Affinity | AF0199 | Rabbit mAb | 1:1000(W.B.) | 130 |
| Arg1 | Affinity | DF6657 | Rabbit mAb | 1:1000(W.B.) | 35 |
| CD86 | Abcam | ab220188 | Rabbit mAb | 1:1000(W.B.) | 38 |
| CD86 | Abcam | ab220188 | Rabbit mAb | 1:200(IF) | 38 |
| CD206 | Abcam | ab64693 | Rabbit mAb | 1:5000(W.B.) | 160 |
| CD206 | Abcam | ab64693 | Rabbit mAb | 1:200(IF) | 160 |
| Bcl-2 | Affinity | AF6139 | Rabbit mAb | 1:1000(W.B.) | 26 |
| Caspase 3 | Affinity | AF6311 | Rabbit mAb | 1:1000(W.B.) | 35 |
| C-Caspase3 | Affinity | AF7022 | Rabbit mAb | 1:500(W.B.) | 17 |
| Bax | Affinity | AF0120 | Rabbit mAb | 1:1000(W.B.) | 21 |
| IκBα | Affinity | AF5002 | Rabbit mAb | 1:1000(W.B.) | 38 |
| p- IκBα | Affinity | AF2002 | Rabbit mAb | 1:1000(W.B.) | 38 |
| NF-κB P65 | Affinity | AF5006 | Rabbit mAb | 1:1000(W.B.) | 65 |
| p38 MAPK | Affinity | AF6456 | Rabbit mAb | 1:1000(W.B.) | 41 |
| p-p38 MAPK | Affinity | AF4001 | Rabbit mAb | 1:1000(W.B.) | 41 |
| ERK1/2 | Affinity | AF0155 | Rabbit mAb | 1:1000(W.B.) | 42/44 |
| p-ERK1/2 | Affinity | AF1015 | Rabbit mAb | 1:1000(W.B.) | 42/44 |
| JNK | Affinity | AF6318 | Rabbit mAb | 1:1000(W.B.) | 46/54 |
| p-JNK | Affinity | AF3318 | Rabbit mAb | 1:1000(W.B.) | 46/54 |
| NeuN | Abcam |  | Rabbit mAb | 1:200(I.F.) | 46 |
| NeuN | Abcam | ab177487 |  | 1:1000(W.B.) | 46 |
| GFAP | Abcam | ab7260 |  | 1:200(I.F.) | 53 |
| GFAP | Abcam | ab7260 |  | 1:1000(W.B.) | 53 |
| Iba-1 | Abcam | ab178846 |  | 1:200(I.F.) | 17 |
| Iba-1 | Abcam | ab178846 |  | 1:1000(W.B.) | 17 |
| CD86 PE | Biolegend | 159203 |  | 1:500 | (Flow cyt) |
| CD206 FITC | Biolegend | 141703 |  | 1:500 | (Flow cyt) |
| CD11bAPC-CY7 | Biolegend | 101225 |  | 1:500 | (Flow cyt) |
| CD45 APC | Biolegend | 147708 |  | 1:500 | (Flow cyt) |
| β-Tubulin | Abcam | ab52623 | Rabbit mAb | 1:50000(W.B.) | 55 |
| Histone H3 | Abcam | Ab1791 | Rabbit PAb | 1:50000(W.B.) | 17 |
| GRPDH | Abcam | ab8245 | Mouse mAb | 1:50000(W.B.) | 35 |
| Beta-Actin | Abcam | ab7817 | Mouse mAb | 1:50000(W.B.) | 42 |

**Supplemental Table 3. Raw DEG Table**
